# POLITAG-M-F as Heterogeneous Organocatalyst for the Waste-Minimized Synthesis of β-Azido Carbonyl Compounds in Batch and under Flow Conditions

**DOI:** 10.1021/acssuschemeng.2c07213

**Published:** 2023-02-08

**Authors:** Federica Valentini, Giulia Brufani, Gabriele Rossini, Filippo Campana, Daniela Lanari, Luigi Vaccaro

**Affiliations:** † Laboratory of Green S.O.C.−Dipartimento di Chimica, Biologia e Biotecnologie, Università degli Studi di Perugia, Via Elce di Sotto 8, 06123 Perugia, Italy, greensoc.chm.unipg.it; ‡ Dipartimento di Scienze Farmaceutiche, Università degli Studi di Perugia, Via del Liceo 1, 06123 Perugia, Italy; # Consorzio Interuniversitario Nazionale per la Scienza e Tecnologia dei Materiali (INSTM), 50121 Firenze, Italy

**Keywords:** β-Azidation reaction, Waste-minimization, Heterogeneous organocatalytic system, Azeotrope, Continuous flow

## Abstract

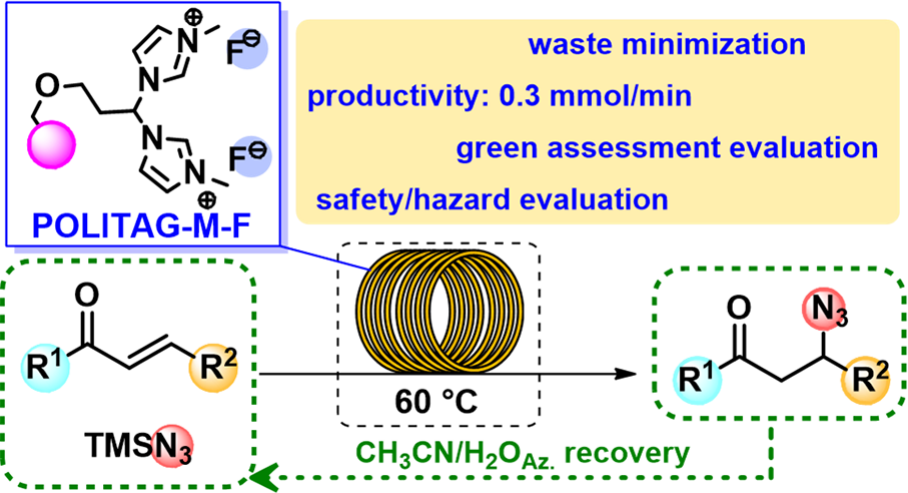

We herein report a waste minimization protocol for the β-azidation of α,β-unsaturated carbonyl compounds using TMSN_3_. The selection of the appropriate catalyst (**POLITAG-M-F**), in combination with the reaction medium, resulted in enhanced catalytic efficiency and a low environmental footprint. The thermal and mechanical stability of the polymeric support allowed us to recover the **POLITAG-M-F** catalyst for up to 10 consecutive runs. The CH_3_CN:H_2_O azeotrope has a 2-fold positive effect on the process, increasing the efficiency of the protocol and minimizing waste generation. Indeed, the azeotropic mixture, used as a reaction medium and for the workup procedure, was recovered by distillation, leading to an easy and environmentally friendly procedure for product isolation in high yield and with a low E-factor. A comprehensive evaluation of the environmental profile was performed by the calculation of different green metrics (AE, RME, MRP, 1/SF) and a comparison with other literature available protocols. A flow protocol was defined to scale-up the process, and up to 65 mmol of substrates were efficiently converted with a productivity of 0.3 mmol/min.

## Abstract 2

POLITAG-M-F is used as a heterogeneous organocatalyst for low environmental footprint continuous flow synthesis of β-azido carbonyl compounds.

## Introduction

Organic azides are known as a well-established class of chemical compounds and valuable synthons in organic chemistry.^1−3^ Thanks to their peculiar reactivity, the azido group plays a crucial role as precursor for amine or amide functional groups, isocyanate via Curtius rearrangement,^4^ and heterocycles^5^ such as 1,2,3-triazoles and tetrazoles via 1,3-dipolar cycloaddition,^6−8^ aza-Wittig reaction,^9,10^ Schmidt rearrangement,^11^ and C–H bond amination.^12,13^

The distinctive reactivity and remarkable biological activity of the azide-moiety have led to a variety of synthetic methodologies for their production. Furthermore, aliphatic azides are common structural units found in a variety of biologically active pharmaceuticals ingredients (API) besides being useful building blocks for the synthesis of a variety of nitrogen-based scaffolds.^14^ For all these reasons, there is an urgent need for the development of innovative and environmentally friendly chemical methods to produce libraries of complex aliphatic azides of special importance.

Azido-Michael reaction of α,β-unsaturated carbonyl compounds represents a key tool for the introduction of novel and complex functionality, forming β-azidocarbonyl compounds which are versatile synthons in organic chemistry.^15−21^

Despite the wide applicability and efficiency of the β-azidation, generally, β-azidocarbonyl compounds are obtained using a combination of sodium azide and a strong acid as hydrazoic acid source,^15−17^ which are unfavorable conditions for the overall safety of the process. For this reason, the development of alternative synthetical strategies involving the use of different sources of azido ion have gained increasing interest. Example of alternative sources is reported by Ramasastry et al. with the development of a metal-free protocol for the β-azidation of α,β-unsaturated ketones using Zhdankin reagent as the azide source and 1,4-diazabicyclo[2.2.2]octane (DABCO) as the catalyst.^18^

Among the azidation agents, trimethylsilyl azide (TMSN_3_) is widely applied in different synthetic approaches for the obtainment of organic azides catalyzed by both organocatalytic systems^19−22^ and metal based catalysts,^23−27^ due to the safer profile of TMSN_3_ in comparison with both hydrazoic acid and sodium azide.^28−30^ A recent example of catalyst free protocol was also proposed but it was limited to the azidation of perfluoroalkyl α,β-unsaturated ketones using TMSN_3_.^31^

From a previously reported full metric evaluation emerged that TMSN_3_ is the best azido source being the best compromise from both the chemical and the environmental points of views among the different azido-sources.^28^ Drawbacks of TMSN_3_ are in all cases just those deriving from the liberation of azido ion. From the evaluation report,^28^ for example, the use of sodium azide negatively affected the overall environmental and safety profile of the azidation process mainly due to its LD50 (oral: 27 mg/kg) and occupational exposure limit. In addition, azides salts when used in the presence of metal catalyst, can form metal azides which are highly dangerous especially when dried.^28^ However, it is worthy to note that also the reaction conditions need to be carefully designed to attain the best safety of a process. Indeed, when TMSN_3_, or any other azides, is used in combination with strong acidic compounds, highly toxic and hazardous HN_3_ can be formed, and therefore, it is essential to adopt conditions that can minimize the HN_3_ formation using catalysts as our solid fluorides, able to rapidly activate the TMSN_3_ but also creating mildly basic conditions.

For these reasons, the full metric evaluation report pointed at the use of TMSN_3_ activated by a recoverable fluoride catalyst as the more benign conditions to thus access a safe and environmentally friendly azidation protocol.^28^

In fact, due to the high affinity of fluoride for silicon that leads to the formation of pentacoordinate complexes, the use of fluoride-based catalysts is usually exploited to generate the azido ion from TMSN_3_.^32,33^ Under these conditions, the formation of hydrazoic acid is avoided, minimizing the risk associated with the process.^28^ Moreover, in the context of a sustainable chemical production, heterogeneous systems, featuring F^–^ ion on solid supports, were also proposed to promote the catalyst recovery and reuse.^34−37^

In the scenario of developing environmentally friendly synthetic protocols, the choice of the reaction medium plays also a pivotal role. β-Azidation processes under Solvent Free Condition (SolFC)^37^ and in water^35^ as reaction medium were previously proposed by our research group in combination with heterogeneous organocatalytic fluoride-based catalysts. Although the reported protocols were associated with low environmental-factor (E-factor)^38^ values and high intrinsic safety, due to the use of water as safe reaction medium or SolFC, the separation of catalyst and product required the addition of extra solvents for the workup procedure, affecting the waste generation.

In agreement with the EPA (Environmental Protection Agency) policies, the recovery of the reaction medium is a critical factor to access waste minimization.^39^ Within our continuous interest in the design of stable and efficient heterogeneous catalytic systems^40−44^ and waste-minimized synthetic protocols, we herein report a low environmental footprint process for the β-azidation of α,β-unsaturated carbonyl compounds using TMSN_3_ as a safe azido source and combining the catalyst design with the selection of the azeotropic mixture CH_3_CN:H_2_O as the recoverable reaction medium.

The use of minimum boiling point azeotropic mixtures allows one to reduce the energy required for the distillation compared to that needed for the distillation of the pure components.^45^ In some cases, the employment of azeotrope as reaction medium enhances the effectiveness of the catalytic system, improving the efficiency of the process.^46−52^

Recently, our research group synthesized a novel class of stable heterogeneous catalyst, namely POLITAG (POLymeric Ionic TAG), made of an ionic tag attached to a polystyrene support and decorated with a counterion (fluoride or iodide) that was successfully employed in SolFC.^53^ Therefore, due the promising performance of POLITAGs, they have been chosen as ideal organocatalytic system to be employed in the β-azidation of carbonyls in azeotropic medium.

Although, it is well-known that ionic moieties covalently bounded to solid polymeric resins may transfer higher stability to the support enhancing the catalyst performance,^54−56^ we selected the 1,4-bis(4-vinylphenoxy)benzene (SPACER) as cross-linker to further improve the **POLITAG-M-F** stability in the reaction medium.^57,58^

The superior performance of our newly developed protocol was also confirmed by a comprehensive evaluation of green metrics^59^—i.e., E-factor, mass recover parameter (MRP), atom economy (AE), reaction mass efficiency (RME) – and their comparison with other available literature protocols through the determination of the vector magnitude ratio (VMR)in radial polygon analysis.^60^

Finally, a continuous-flow protocol was proposed aiming at minimizing the environmental footprint of the process and improving catalyst durability.^61^

## Results and Discussion

The different organocatalytic systems, namely POLITAGs, tested in this work, were obtained by varying the ratio between the comonomers (styrene and 4-vinylbenzyl chloride) during the suspension copolymerization, using 2% of SPACER as cross-linker (see Supporting Information). This procedure afforded different loaded gel-type polymeric supports, namely SP02-M and SP02-L, where M (medium) and L (low) are referred to the loading of Cl on their surface.

The differences in the Cl amount on the heterogeneous supports are reflected in a different loading of active catalytic sites after the postpolymerization functionalization steps, crucial for the selection of the proper catalytic system.

After nucleophilic substitution reaction between the chloromethyl functionalized resins and the 3,3-bis(1*H*-imidazol-1-yl)propan-1-ol ligand,^62^ the quaternization reaction of the obtained material in the presence of iodomethane gives the polymer-supported bis(imidazolium)-based organocatalysts **POLITAGs-I**, where I indicates the iodide counterion (see Supporting Information).

**Scheme 1:**
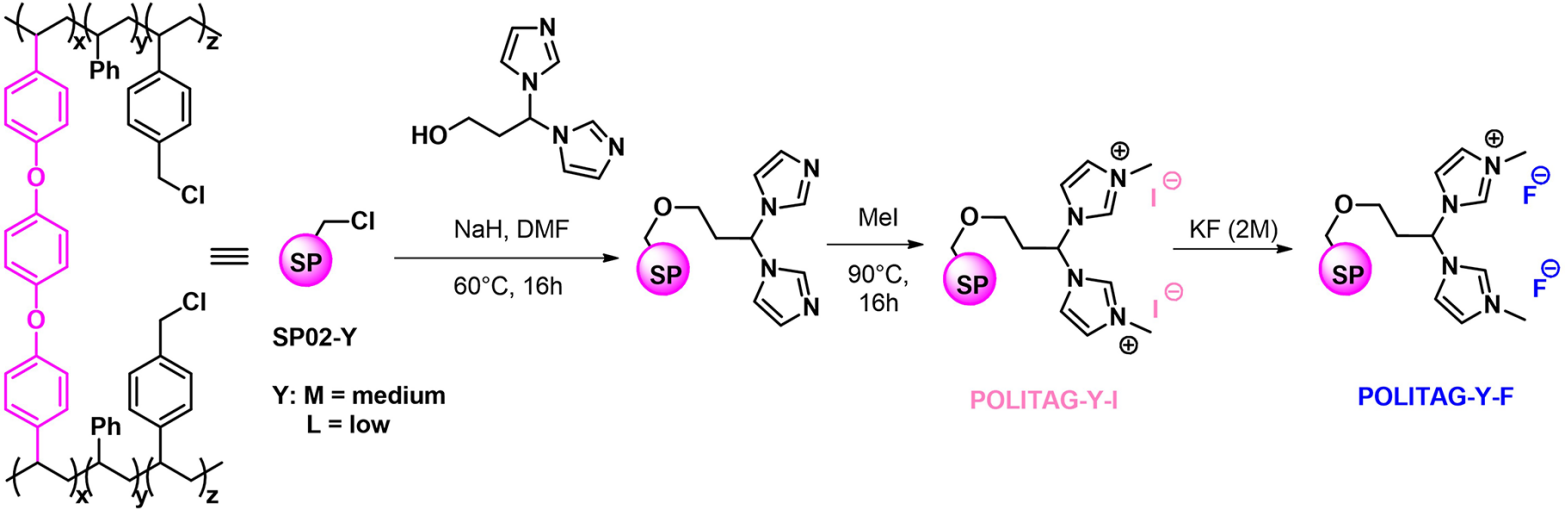
Schematic Representation of POLITAGs Synthesis

The corresponding fluorinated materials **POLITAGs-F** have been efficiently obtained by ionic exchange in the presence of KF aqueous solution (2 M) until no silver iodide precipitates from the eluted solution as confirmation of the quantitative ion exchange (Scheme 1). The efficiency of the above-described procedure was previously confirmed by several solid-state characterization techniques.^53^

The loadings of I^–^ and F^–^ anion were determined by elemental analysis for both medium and low catalyst loadings (see Supporting Information for further details).

The prepared catalysts **POLITAGs** were tested in the representative β-azidation reaction of (*E*)-3-hepten-2-one (**1a**) using TMSN_3_ as an azido source (see scheme of Table 1).

**Table 1:**
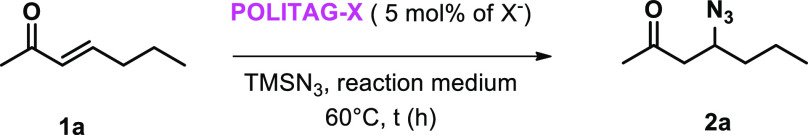
Optimization of Reaction Conditions for β-Azidation of **1a** to **2a**^a^

entry	catalyst	reaction medium	conc (M)	*t* (h)	conv (%)^b^
1	POLITAG-L-F	SolFC	–	8	43
2	POLITAG-M-F	SolFC	–	8	31
3	POLITAG-M-F	CH_3_CN	2	2.5	50
4	POLITAG-M-F	CH_3_CN:H_2_O_Az._	2	2.5	84
5	POLITAG-M-F	CH_3_CN:H_2_O_Az._	4	2.5	91
6	POLITAG-M-F	CH_3_CN:H_2_O_Az._	5	2.5	97^c^
7	POLITAG-L-I	CH_3_CN:H_2_O_Az._	5	2.5	31^c^
8	POLITAG-M-I	CH_3_CN:H_2_O_Az._	5	2.5	40^c^
9	POLITAG-L-F	CH_3_CN:H_2_O_Az._	5	2.5	87^c^
10	POLITAG-M-F	CH_3_CN:H_2_O_Az._	10	2.5	97^c^

a Reaction condition: **1a** (1 mmol), TMSN_3_ (1.1 equiv), **POLITAG-X** (5 mol % of X^–^), reaction medium (M), 60 °C.

b Conversion determined by GC analysis. The remained material is unreacted **1a**.

c Reactions performed with TMSN_3_ (1.05 equiv),

When the reaction was performed in SolFC poor results were obtained with both POLITAGs-F catalysts (entries 1–2, Table 1). Although under these conditions the **POLITAG-L-F** system gave better results compared with the **POLITAG-M-F** catalyst, the conversion after 8 h of reaction time was not satisfactory. The better performance of low loading catalyst under SolFC was not surprising, and it is mainly ascribable to a widely availability of the catalytic site on polymer surface. Indeed, when high ionic moieties are present on gel-type support, an ionic association, hindering the catalytic efficiency, is likely to occur. On the contrary, when the material is placed in a good swelling solvent, all the catalytic sites are accessible and a better comparison could be achieved for the selection of the appropriate catalyst.

Moreover, the heterogeneous catalyst employed under SolFC makes difficult the stirring of the reaction mixture. For these reasons, we decide to test our **POLITAG-M-F**, in order to minimize the mass of catalyst needed, in CH_3_CN and CH_3_CN:H_2_O azeotropic mixture as reaction media. When the reaction was performed in acetonitrile a 50% of product **2a** was obtained after 2.5 h (entry 3, Table 1) instead of 31% afforded after 8 h under SolFC (entry 2, Table 1). This conversion was further improved by using the CH_3_CN:H_2_O azeotropic mixture that allowed to reach an 84% of product **2a** (entry 4, Table 1). This superior result could be easily explained considering the components of the azeotropic mixture: as demonstrated in a previous work,^35^ water plays a positive influence affecting the reaction mechanism, while CH_3_CN was crucial to promote the swelling of the gel-type resin ensuring the availability of the active sites (entry 4, Table 1).

With the selected reaction medium, we investigated the effect of the concentration to minimize the use of solvent and to find the best compromise between the polymer swelling and the stirring of the reaction mixture (entries 4–6 and 10, Table 1). By increasing the concentration to 5 and 10 M was also possible to reduce the equivalent of TMSN_3_ needed to 1.05 equiv instead of 1.1 equiv obtaining a conversion in **2a** of 97% (entries 6 and 10, Table 1).

We selected 5 M aqueous acetonitrile azeotrope to compare the effect of catalyst loading and counterion. By using **POLITAG-L-I** and **POLITAG-M-I**, the conversion was not satisfying (entries 7–8, Table 1), due to the trimethylsilyl iodide formation which may also decompose in the presence of water forming HI. Good results were observed only when fluoride based POLITAGs were used affording the best result with the medium loading catalyst.

To confirm the role of the polymeric support in the catalytic efficiency we decided to synthesize additional heterogeneous fluoride-based catalysts with commercially available polymeric supports and test the material in the β-azidation of **1a** (Table 2). The catalysts **M-F** and **JJ-F**, obtained by immobilizing the bis-imidazolium ionic-tag on Merrifield and JandaJel resins respectively, despite giving good conversion in product **2a** (entries 3 and 4, Table 2), were less efficient in comparison with our **POLITAG-M-F** catalyst confirming the importance of the SPACER cross-linker. In particular, the Merrifield resin has divinylbenzene (DVB) as a small and rigid cross-linker, while the JandaJel support is a highly flexible solvent-like polymer featuring a polytetrahydrofuran-based (PTHF) cross-linker.

**Table 2:**
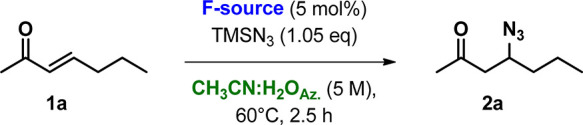

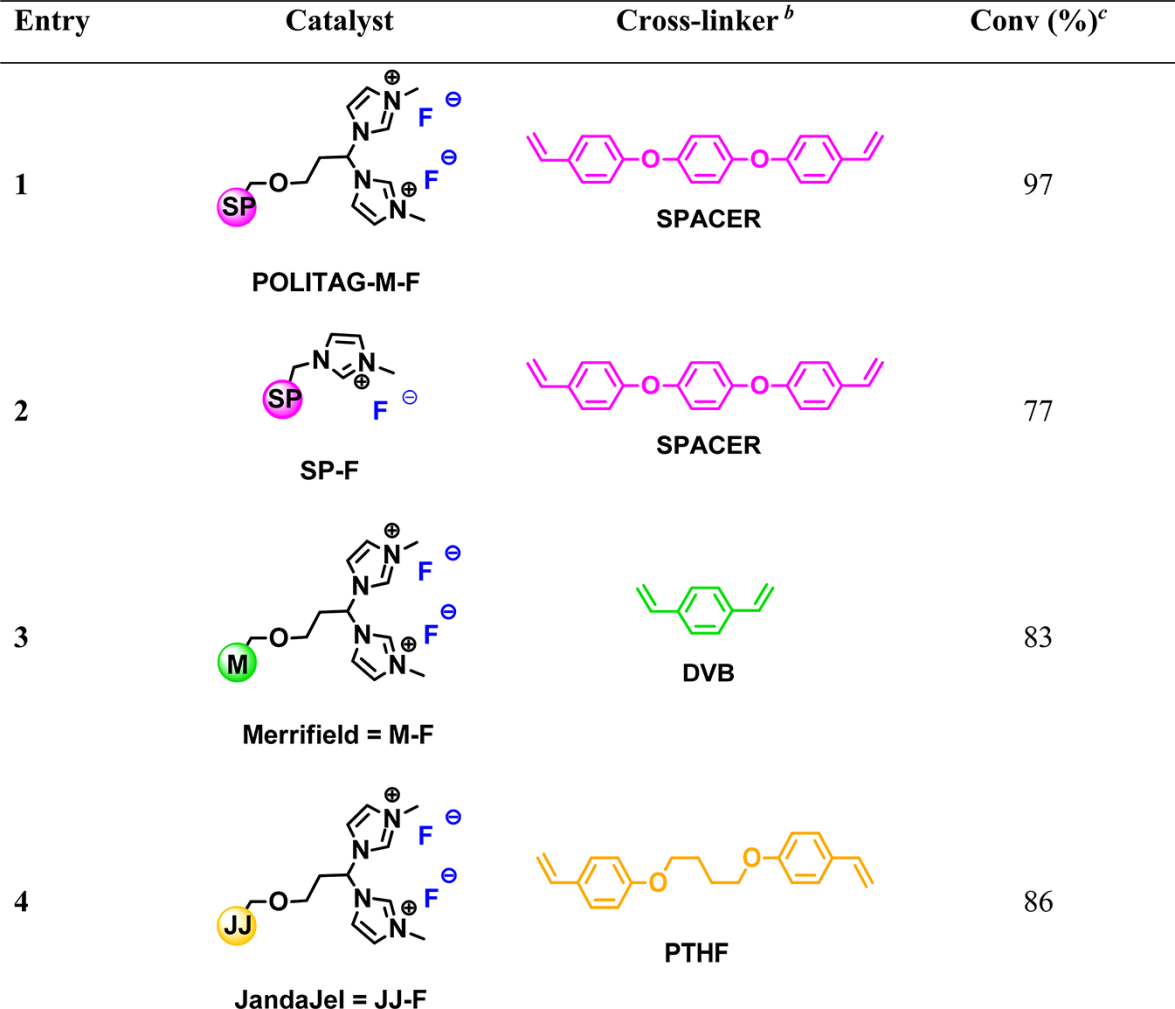
Screening of our Different Heterogenous Sources of Fluoride in the β-Azidation of **1a** to **2a**^a^^c^

a Reaction conditions: **1a** (1 mmol), TMSN_3_ (1.05), fluoride sources (5 mol % of F^–^), CH_3_CN:H_2_O_Az._ (5 M), 60 °C.

b DVB = divinylbenzene; PTHF = polytetrahydrofuran based cross-linker.

c Conversion determined by GC analysis. The remaining material is unreacted **1a**.

A third additional system was obtained by the immobilization of imidazolium on SP02-M gel-type polymeric support affording **SP-F** catalyst, the lower conversion (entry 2, Table 2) compared with the other catalytic systems highlights the importance of the pincer type ionic-tag structure.

Other commercially available fluoride-based catalysts were also tested in the β-azidation of **1a** under the optimized reaction conditions (Table 3). Among the system tested only homogeneous tetrabutylammonium fluoride (TBAF) showed comparable results with our **POLITAG-M-F** (entry 1, Table 3), immediately followed by TBAF supported on silica gel (entry 5, Table 3).

**Table 3:**
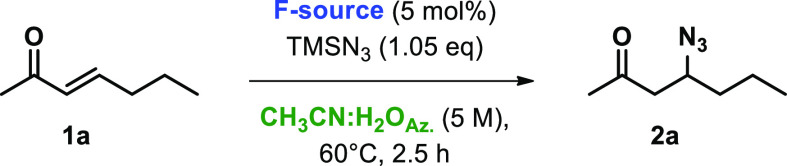
Screening of Different Sources of Fluoride in the β-Azidation of **1a** to **2a**^a^

entry	catalyst	conv (%)^b^
1	TBAF	97
2	Amberlite IRA400F^–^	82
3	Amberlite IRA900F^–^	90
4	KF on alumina	88
5	TBAF on silica gel	95

a Reaction conditions: **1a** (1 mmol), TMSN_3_ (1.05), F-sources (5 mol %), CH_3_CN:H_2_O_Az._(5 M), 60 °C.

b Conversion determined by GC analysis. The remaining material is unreacted **1a**.

Even if this latter showed better performances compared with gel-type catalyst Amberlite IRA400F^–^ (entry 2, Table 3), macroreticular catalyst as Amberlite IRA900F^–^ (entry 3, Table 3), and KF on alumina (entry 4, Table 3), after the recovery and reuse, the catalytic efficiency dropped affording 88% of conversion in product **2a**.

On the contrary, **POLITAG-M-F** was recovered and reused in the β-azidation reaction for more than 10 consecutive runs without any loss in efficiency (see Supporting info, Figure S1).

**Scheme 2:**
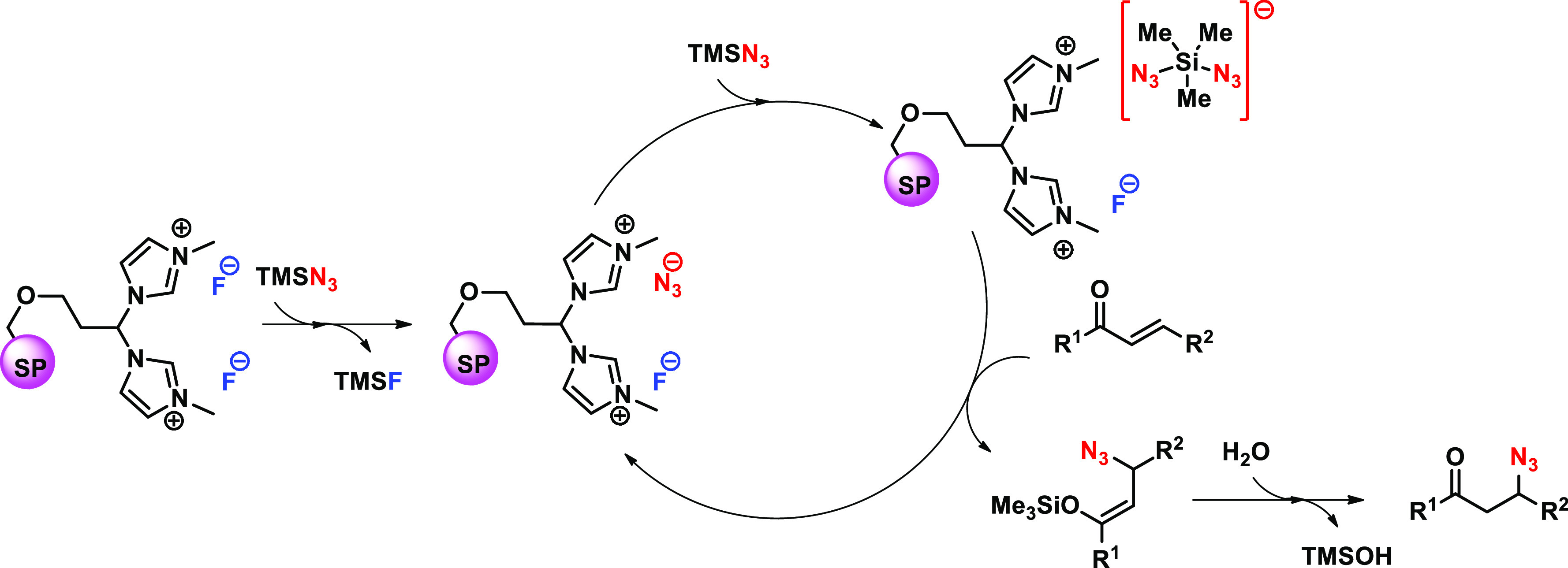
Proposed Mechanism for POLITAG-F-Catalyzed β-Azidation Reaction of α,β-Unsaturated Carbonyl Compounds

The elemental analysis of the recovered **POLITAG-M-F** exhibits an increased quantity of N compared to that of fresh **POLITAG-M-F** (Fresh: C, 68.64; N, 7.43; H, 8.353. Recycled: C, 66.53; N, 9.13; H, 7.681.), suggesting a partial exchange of fluoride counterion with azido ion during the catalytic cycle,^37^ which does not affect the efficiency of the catalytic system. Considering this evidence, we hypothesized the POLITAG having N_3_^–^ anion as the active catalyst (Scheme 2). The latter forms, in the presence of TMSN_3_, a pentavalent silicon complex able to attack the α,β-unsaturated carbonyl compound leading to the 1,4-addition product which rapidly hydrolyzed in the presence of water. Under these conditions TMSOH byproduct and the activated POLITAG catalyst were formed.

The effectiveness of our carefully designed **POLITAG-M-F** in the β-azidation reaction of α,β-unsaturated carbonyl compounds was compared, in term of TON and TOF, with other heterogeneous and homogeneous catalysts (Table 4). The high durability of our catalyst for several consecutive runs allowed us to obtain superior TON and TOF values.

**Table 4:** TON and TOF Comparison for **POLITAG-M-F** and other Heterogenous and Homogenous Catalysts in the β-Azidation Reaction

catalyst	TON	TOF	ref
(Salen)Al complex	35.2	1.5	(15)
peptide catalyst	40	1.6	(19)
PS-DABCOF_2_^a^	49.5	4.0	(35)
Amberlite IRA900F^b^	45	3.6	(37)
POLITAG-M-F^a^	194	7.8	this work

a Recovered and reused for 10 consecutive runs.

b Recovered and reused for 5 consecutive runs.

**Figure 1:**
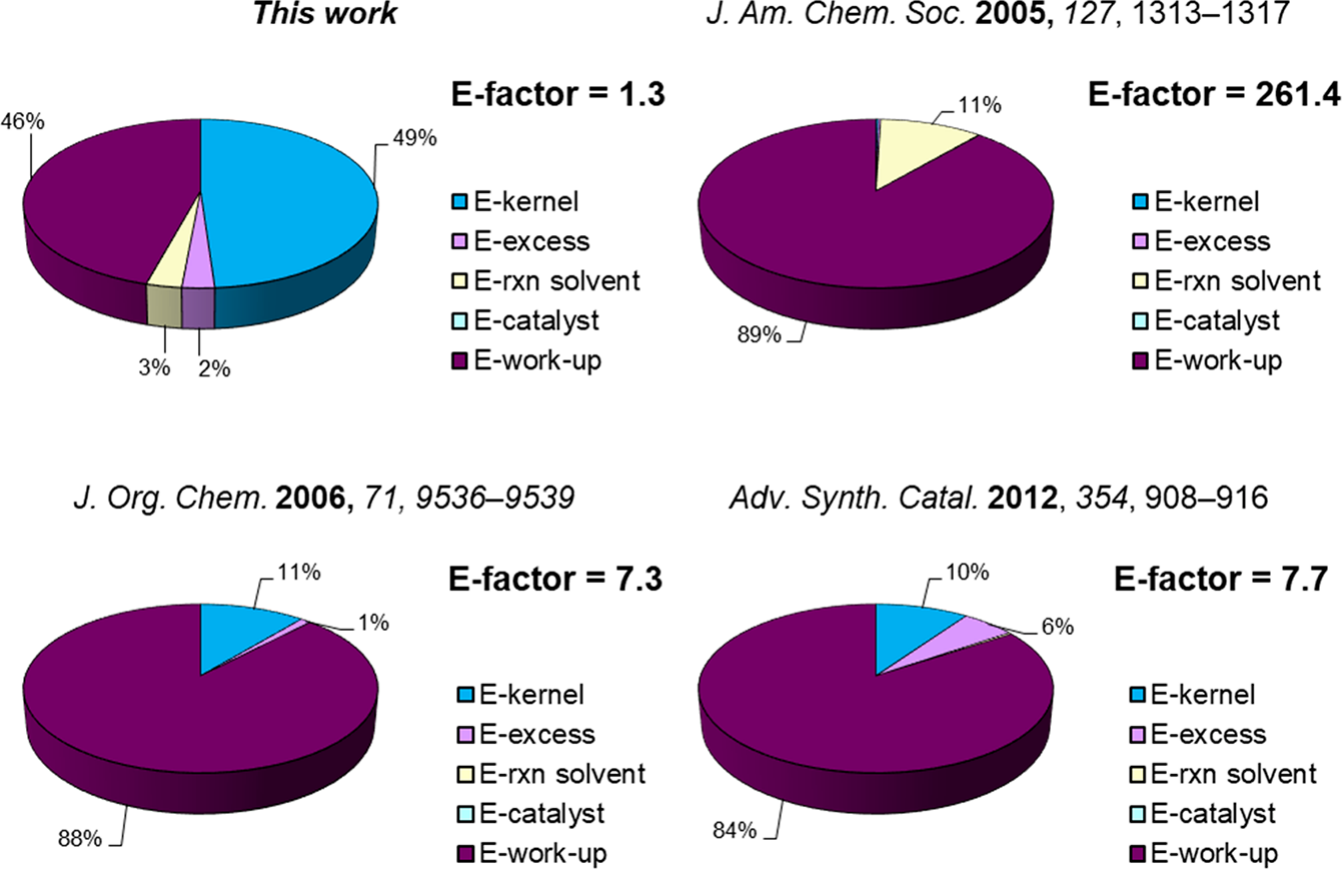
Comparison of the E-factor distribution for the β-azidation reaction of **1a**.

**Scheme 3:**
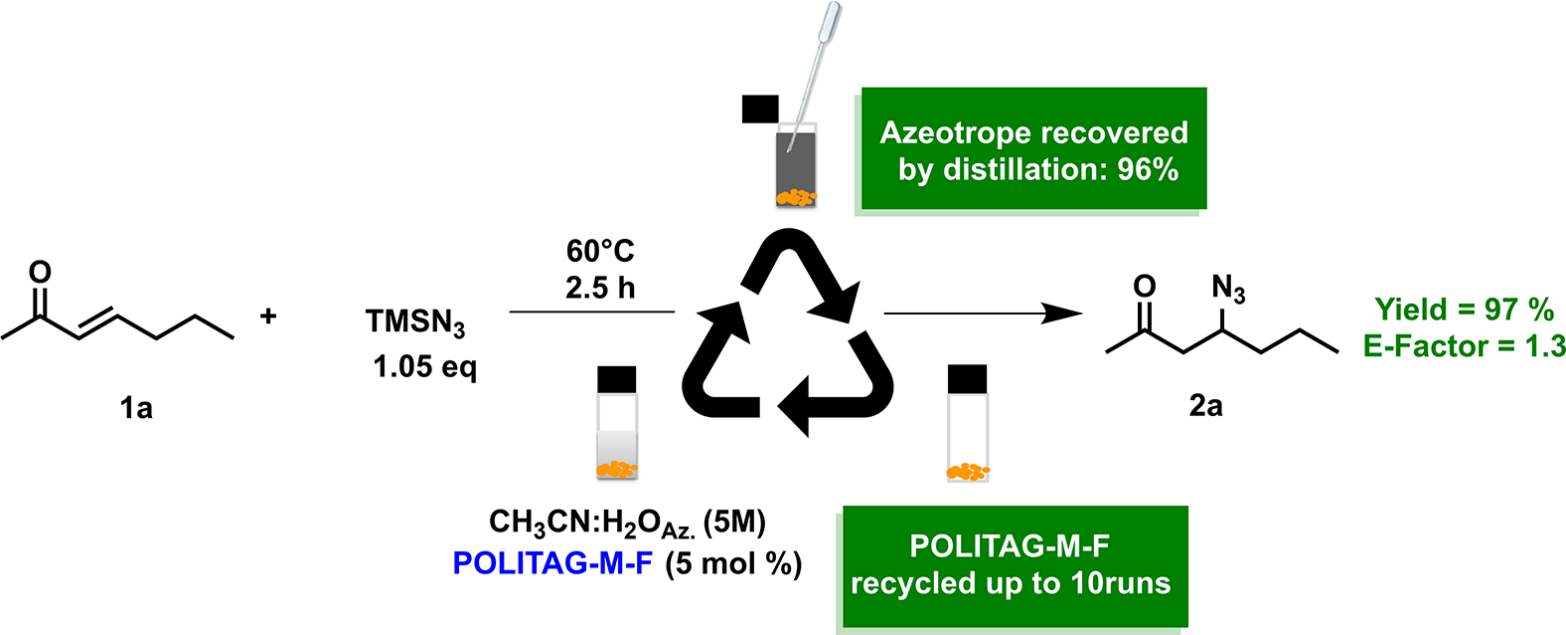
Recycle of POLITAG-M-F and Azeotrope CH_3_CN:H_2_O

It is worth noticing that the CH_3_CN:H_2_O azeotrope, used as reaction medium and in the workup procedure, was recovered at 96% via distillation affording 97% isolated the pure product **2a** (Scheme 3). The catalyst and azeotrope recyclability led to the definition of efficient and waste-minimized procedure with an E-factor value of 1.3 (Figure 1). This value is significantly lower if compared with other literature available protocols leading to product **2a** (see Figure 1 and Supporting Information for further details).^15,35,37^ Indeed, also when the reaction was performed in SolFC,^37^ the necessity of additional solvents to separate product **2a** from the catalyst negatively affected the E-factor. The workup is the main contribution to the E-factor for all the selected protocols (84–89%); while by considering the E-factor profile of herein developed process this contribution was reduced to 46%.

Moreover, the E-factor value was further reduced by conducting the process on 10 mmol scale. The high yield obtained under these conditions (99%) proved the scalability of our protocol affording an E-factor of 0.7.

**Scheme 4:**
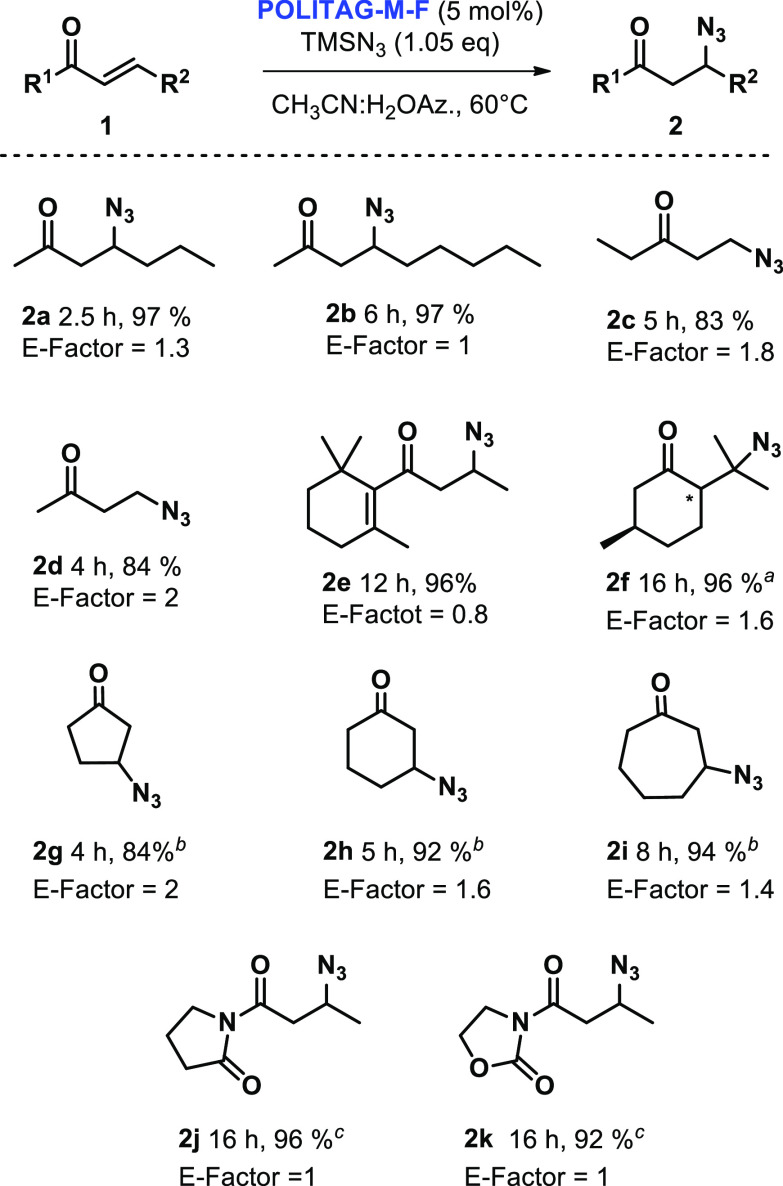
β-Azidation of Different α,β-Unsaturated Carbonyl Compounds Catalyzed by **POLITAG-M-F** in CH_3_CN:H_2_O TMSN_3_ (2.0 equiv), **POLITAG-M-F** (12.5 mol % of F^–^ ion); CH_3_CN:H_2_O_Az._ (10 M). TMSN_3_ (1.2 equiv), **POLITAG-M-F** (12.5 mol % of F^–^ ion), CH_3_CN:H_2_O_Az._ (10 M). **POLITAG-M-F** (10 mol % of F^–^ ion), CH_3_CN:H_2_O_Az._ (10 M). Reaction conditions: **1** (1 mmol), TMSN_3_ (1.05 equiv), **POLITAG-M-F** (5 mol % of F^–^ ion), CH_3_CN:H_2_O_Az._ (5 M), 60 °C.

Considering the low-environmental impact of the developed protocol, it was interesting to test the efficiency of the **POLITAG-M-F** and aqueous acetonitrile azeotrope in the fluoride-catalyzed β-azidation reaction of different α,β-unsaturated carbonyl compounds (Scheme 4).

When linear α,β-unsaturated carbonyl compounds reacted with TMSN_3_ (1.05 equiv) products **2b**−**e** were obtained with good to excellent isolated yields. The reaction of β-damascone (**1e**) was completely regioselective in yielding product **2e**; while the reaction of (*R*)-(+)-pulegon (**1f**) required an increased amount of TMSN_3_ (2.0 equiv) to give product **2f** in 96% yield in a 60/40 mixture of two diastereoisomers. Cyclic substrates **1g**–**i** required an increment of **POLITAG-M-F** amount and a slight excess of TMSN_3_ to afford products **2f**–**i** in isolated yield in the range of 84–94%. Pyrrolidinone (**2j**) and oxazolidinone (**2k**) products were also obtained in excellent yields (Scheme 4).

These results confirmed that the synthesized heterogeneous fluoride-based organocatalyst is an effective catalytic system for the β-azidation reaction of different α,β-unsaturated carbonyl compounds using CH_3_CN:H_2_O azeotrope as recoverable reaction medium. Very low E-factor values, ranging from 0.8 to 2.1, were calculated for the products obtained (for E-factor calculation see Supporting Information).

**Figure 2:**
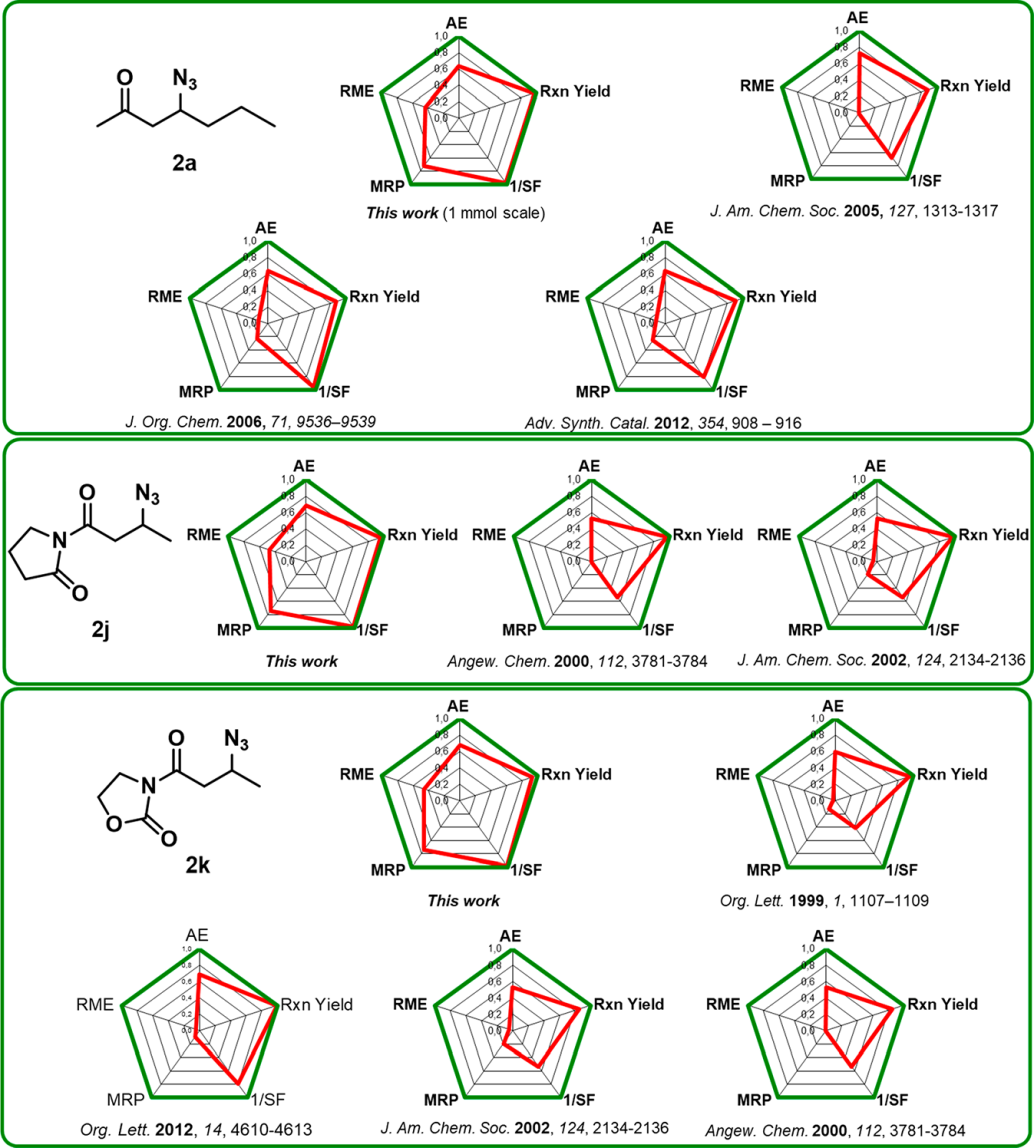
Radial polygons for substrates **2a**, **2j**, and **2k** of our protocol and comparison with literature available procedures.

For a deeper comprehension of the sustainability of our protocol we evaluated different green-metrics (MRP, AE, SF and RME) selecting three products (**2a**, **2j**, and **2k**) and compared our process with other literature available batch protocols (Figure 2 and Table S2).

The choice of azido-source, as well as the use of acidic additives, is reflected in the Atom Economy (AE) metric. Indeed, when the β-azidation reaction of **1a** to **2a** is performed with sodium azide^15^ instead of TMSN_3_, a higher AE value was obtained (0.726 vs 0.632). It is worth noticing that an equimolar amount of water was considered for the calculation of the metrics since it is consumed in stoichiometric amounts in agreement with the reaction mechanism (Scheme 2). On the contrary, when acids are employed in combination with TMSN_3_,^20−22^ the AE value dropped to 0.600 and 0.530 if acetic acid^22^ or pivalic acid^20,21^ are used respectively.

The possibility to recover the catalyst and the azeotropic aqueous acetonitrile mixture, used as reaction medium and for the workup procedure, positively affected the environmental profile of the developed protocol approaching (red line) the ideal area of the radial polygon (green line). In particular, the mainly influenced metrics are the Mass Recover Parameter (MRP) and the Reaction Mass Efficiency (RME).

In addition, we also evaluated the environmental impact of the azido-source, TMSN_3_ (this work) and NaN_3_,^15^ by calculating the Safety Hazard Index (SHI)^63^ and Benign Index (BI)^64^ for both input and waste materials (see Supporting Information for further details).

We can notice that SHI and BI for the input material give apparent similar results (see Table S8 in the Supporting Information). More in details, it can be noticed that hazard and environmental parameters are generally and significantly worse for NaN_3_ compared to TMSN_3_. The final similarity is due to the lower MW of NaN_3_ that influenced the weighted impact potential. Anyway, the occupational exposure limit potential (OELP) for NaN_3_ associates this chemical with a much higher risk.

When the environmental impact is calculated on the waste materials (see Table S8 in the Supporting Information), the results do not require additional explanations. In the case of our procedure a very high BI (0.9260) close to the ideal situation was calculated (BI value ranges from 0 to 1, with 1 being the ideal situation). In the case of NaN_3_ the benign index of the mixture is as low as 0.5959. As an additional comment, in this latter case, we have also omitted the additional formation of highly toxic and hazardous HN_3_ (formed from NaN_3_ in the presence of HCl) since it would lead to an even worse environmental and safety profile when using NaN_3_.

The higher risk for health and environment related to NaN_3_ is also finally corroborated by the GSH (Globally Harmonized System) Hazard Classification which assigns a category 1 for dermal toxicity (while TMSN_3_ is 3).

**Scheme 5:**
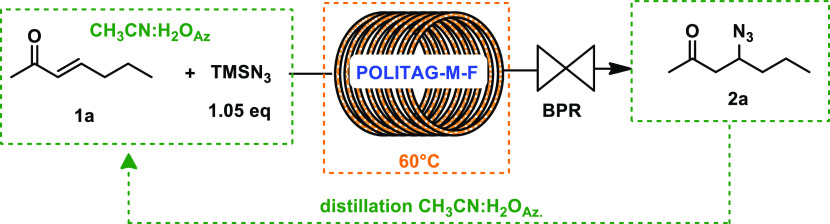
Flow Protocol Developed for β-Azidation of **1a** to **2A** Using **POLITAG-M-F**

Thanks to the durability of our **POLITAG-M-F** catalyst, we decided to further improve the sustainability of our protocol developing a continuous flow process (Scheme 5).

The design of the reactor was crucial for the optimization of flow protocol (Table S1 in the Supporting Information). Reducing the internal diameter (ϕ) and increasing the reactor length, in the presence of BPR of 10 atm, allowed us to adjust the residence time (40 min) and obtain the optimized conditions (entry 8, Table S1).

A complete conversion of **1a** to **2a** was achieved by continuously fluxing the mixture of (*E*)-3-heptene-2-one (**1a**) and TMSN_3_ (1.05 equiv) in CH_3_CN:H_2_O azeotrope through the reactor packed with a mixture of **POLITAG-M-F** (947 mg) and quartz powder thermostated at 60 °C (see Supporting Information for further details).

The optimized protocol allowed to continuously convert 50 mmol of **1a** in short reaction time (<3 h) with a productivity of 0.3 mmol/min representing an important advantage in the scalability of the proposed protocol.

After the elution of the reaction mixture, the line and the reactor were cleaned with acetonitrile aqueous azeotrope. The desired product **2a** was easily isolated in high yield (99%) after the recovery of the azeotropic mixture by distillation.

**Figure 3:**
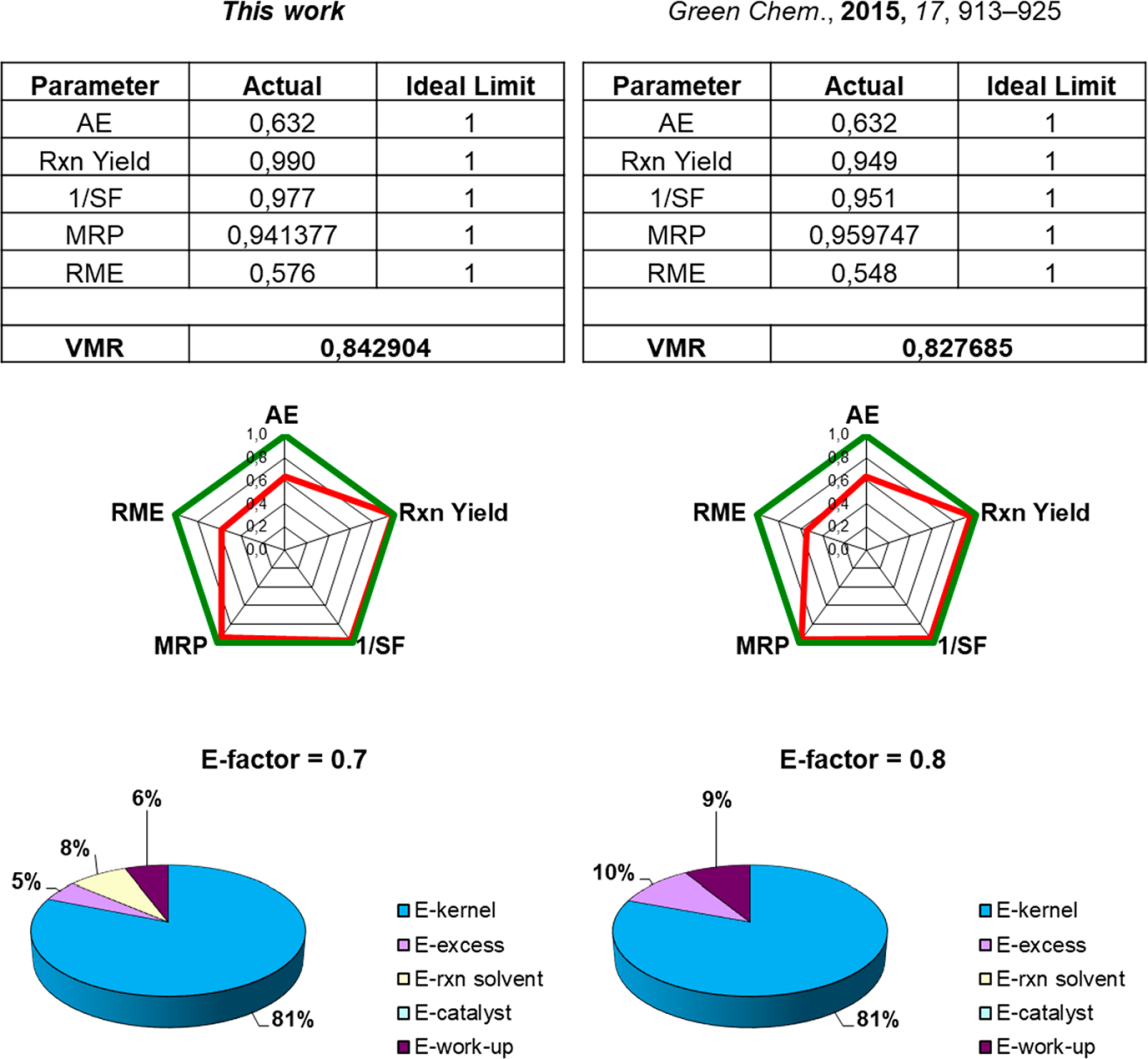
Comparison of green metrics in β-azidation reaction of **1a** under continuous flow protocol.

At this point, we evaluated the improvements of the herein developed flow protocol by the comparison of different green metrics (Figure 3) with the continuous flow process previously proposed under SolFC.^28^ This latter showed until now the lowest E-factor value and good VMR due to high 1/SF and MRP. These values were certainly ascribable to the avoidance of reaction medium utilization and to the recovery of the solvent used for the workup.^28^ However, the high **POLITAG-M-F** efficiency in the selected CH_3_CN/H_2_O azeotrope led to an increase in the environmental profile of the reaction reducing the E-factor of 12.5% (0.7 vs = 0.8) and slightly increasing the VMR. The main gain of this protocol is the utilization, and the subsequent recovery, of the azeotropic mixture as well as the minimization of excess of TMSN_3_.

**Scheme 6:**
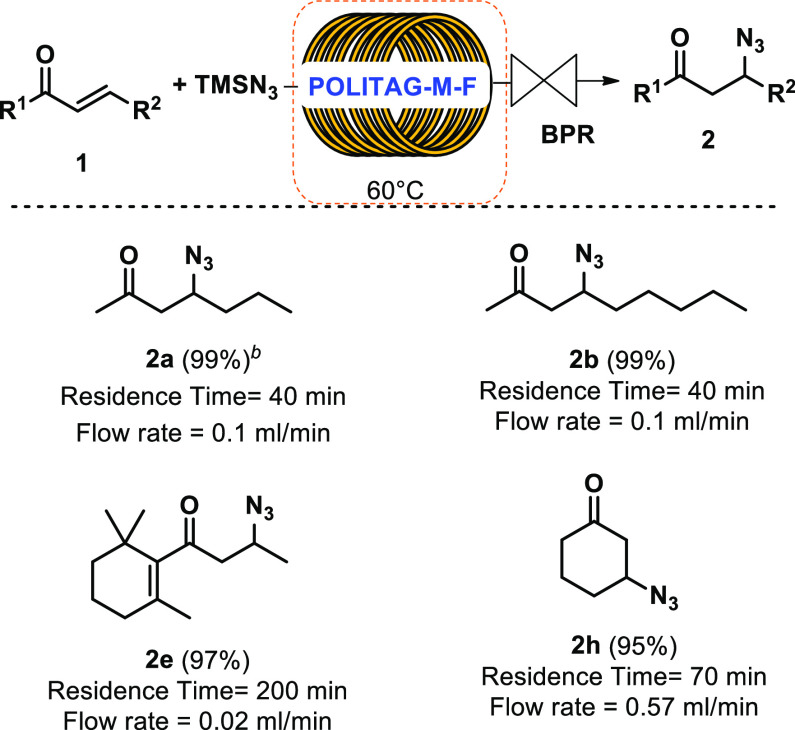
β-Azidation of Different α,β-Unsaturated Carbonyl Compounds under Continuous Flow Conditions Reaction conditions: **1** (5 mmol), TMSN_3_ (1.05 equiv), CH_3_CN/H_2_O_Az._ (5 M), BPR (10 atm), 60 °C. Reaction performed on 50 mmol scale.

The efficiency of the developed flow protocol was then extended to other α,β-unsaturated carbonyl compounds using the same packed reactor (Scheme 6). Excellent isolated yields were obtained for different substrates by adjusting the flow rate and the residence time.

The designed reactor, packed with **POLITAG-M-F**, converted up to 65 mmol of α,β-unsaturated carbonyl compounds in β-azido carbonyl compounds without showing any decrease in the catalytic efficiency.

## Conclusion

The waste-minimized β-azidation protocol of α,β-unsaturated carbonyl compounds was developed by carefully combining the choice of reaction medium and heterogeneous **POLITAG-M-F** catalyst. Indeed, both the selection of catalyst loading and counterion played a fundamental role in the reported process. Moreover, the influence of the cross-linker and the ionic moiety were investigated, and the catalytic efficiency was compared with that of commercially available fluoride-based catalysts. In addition, the elemental analysis of fresh and used **POLITAG-M-F** suggested an ion exchange between fluoride and azido anions during the catalytic cycle.

The selection of CH_3_CN:H_2_O azeotrope as reaction medium enhanced the conversion of α,β-unsaturated carbonyl compounds minimizing the waste associated with the process. The products were easily isolated in high yields removing the low boiling point azeotrope by distillation leading to very small E-factor values ranged from 0.8 to 2.1.

The selected POLITAG-M-F catalyst showed impressive durability being recycled for up to 10 consecutive runs without showing any decrease in the catalytic efficiency.

The utilization of recoverable catalyst-reaction medium system is the main parameter that contributed to highly enhance the sustainability of the process as demonstrated by the analysis of different green metrics (AE, MRP, RME, and 1/SF), VMR and the comparison with literature available protocols.

Furthermore, the catalyst showed great stability and high efficiency also under flow condition, allowing to convert up to 65 mmol of different β-azido carbonyls with high productivity (0.3 mmol/min). To the best of our knowledge, the flow protocol developed is associated with the lowest E-factor value in literature for the selected process (0.7).

## Supporting Information Available

Experimental procedures, compound characterizations, and copies of ^1^H and ^13^C spectra (PDF)

## Abbreviations

POLITAG-M-F: polymeric ionic tag, with medium loading fluoride as counterion
SolFC: solvent free condition
TBAF: tetrabutylammonium fluoride
BPR: back pressure regulator
SP-F: polymeric support, ligand imidazolium based with fluoride as counterion
M-F: Merrifield functionalized with bis(imidazolium) ionic tag with fluoride as counterion
JJ-F: JandaJel functionalized with bis(imidazolium) ionic tag with fluoride as counterion
MRP: mass recovery parameter
RME: reaction mass efficiency
AE: atom economy
SF: stoichiometric factor
