# Supplementary material for: POLITAG-M-F as Heterogeneous Organocatalyst for the Waste-Minimized Synthesis of β-Azido Carbonyl Compounds in Batch and under Flow Conditions
Source: ACS Sustain Chem Eng. 2023 Feb 8;11(7):3074–84. doi: 10.1021/acssuschemeng.2c07213 (PMC9945162; doi:10.1021/acssuschemeng.2c07213)

SUPPORTING INFORMATION FILE

# POLITAG-M-F as heterogeneous organocatalyst for the waste minimized synthesis of β-azido carbonyl compounds in batch and under flow conditions.

Federica Valentini,[‡a] Giulia Brufani,[‡a] Gabriele Rossini,[a] Filippo Campana,[a,c] Daniela Lanari,[*b] Luigi Vaccaro[*a]

[a]*Laboratory of Green S.O.C. – Dipartimento di Chimica, Biologia e Biotecnologie, Università degli Studi di Perugia, Via Elce di Sotto 8, 06123 – Perugia, Italy. Web: greensoc.chm.unipg.it*

[b]*Dipartimento di Scienze Farmaceutiche, Università degli Studi di Perugia, Via del Liceo 1, 06123 – Perugia – Italy*

[c]*Consorzio Interuniversitario Nazionale per la Scienza e Tecnologia dei Materiali (INSTM) Via Giusti, 9, 50121 Firenze (Italy)*

[‡]Both authors contributed equally to this work.

e-mail: luigi.vaccaro@unipg.it
daniela.lanari@unipg.it

total number of pages: 35

total number of figures: 1 at page S5

total number of tables: 8 at pages S5 and S11-S13

Table of contents:

1. General remarks S2

2. General procedures S3

3. E-factor calculations and green assessment analysis S6

4. Benign Index and Safety Hazard Index data S12

5. Characterization data S14

6. $^{1}H$ and $^{13}C$ spectra S25

## 1. General Remarks

4-Vinylbenzylchloride (VBC) and styrene (STY) were extracted three times with a 10% w/w NaOH solution and filtered through $Al_2O_3$ pad to remove the polymerization inhibitor (*tert*-butyl catechol). Dibenzoylperoxide was re-crystallized from methanol. Trimethylsilyl azide (97% pure, d: 0.860 Kg/L) and β-azido ketones were purchased from Sigma-Aldrich, Alfa Aesar and TCI. GLC analyses were performed by using Agilent 5890 Series II GC System equipped with a capillary column DB-5MS (30 m, 0.32 mm), a FID detector and helium as gas carrier. GC-EIMS analyses were carried out by using a Hewlett-Packard HP 6890N Network GC system/5975 Mass Selective Detector equipped with an electron impact ionizer at 70 eV. All $^{1}$H-NMR, $^{13}$C-NMR spectra were recorded on a Bruker DRX-ADVANCE 400 MHz ($^{1}$H at 400 MHz and $^{13}$C at 100.6 MHz) using a convenient deuterated solvent ($CDCl_3$). Chemical shifts are reported in ppm (δ) and coupling constants (J) in Hertz and multiplicity are reported as follows: *s* = singlet, *bs* = broad singlet, *d* = doublet, *dd* = double doublet, *td* = double triplet, *t* = triplet, *m* = multiplet. Spectra are reported considering the product purity >95%. Thin Layer Cromatography analyses were performed on silica gel on silica plates and UV and/or $KMnO_4$ were used as revealing systems.

Compound **1j** and compound **1k** were synthesized according to literature available procedure.[1]

3,3-bis(1H-imidazol-1-yl)propan-1-ol ligand[2] and SPACER cross-linker[3] were prepared according to previously reported procedures.

Characterization data and copies of the $^{1}$H and $^{13}$C NMR spectra of the compounds are reported below.

***Caution!*** All the procedures should be carried out in a fume hood wearing personal protective equipment. Detailed information regarding the safety hazards of all chemicals should be obtained from the Safety Data Sheets (SDS).

## 2. General Procedure

*Synthesis of **SP02-Cl** supports*.

A three-neck cylinder-shaped glass vessel, equipped with a mechanical stirrer, a condenser, and a nitrogen inlet was charged with 120 mL of an aqueous NaCl (4 wt%, 4.8 g) solution containing acacia gum (8 wt%, 9.6 g). After deoxygenation, a mixture of styrene (L: 5.62 g, 53.95 mmol; M: 2.98 g, 28.6 mmol), vinylbenzylchloride (L: 1.48 g, 9.75 mmol; M: 5.36 g, 35.1 mmol), 1,4-bis (4-vinylphenoxy)benzene (0.409 g, 1.3 mmol), chlorobenzene (8 mL), and dibenzoylperoxide (2% wt/wt, 150 mg, 0.62 mmol) was rapidly added to the solution. The suspension was kept under mechanical stirring at room temperature for an additional 30 min, then heated at 85 °C for 24 h. Subsequently, the reaction mixture was cooled and the polymer beads were isolated by filtration, washed with water (50 mL), then extracted by using water (100 mL), THF (100 mL), and n-hexane (100 mL) in a Soxhlet apparatus.

The resulting SP02-Cl resins were dried under vacuum for 15 h at room temperature. The amount of Cl on polymer surface was determined by elemental analysis (L: 1.4 mmol/g, Cl: 5.055; M: 3.4 mmol/g, Cl: 12.078).

*Synthesis of **POLITAGs-I.***

A 25 mL two-neck round bottom flask equipped with magnetic stirrer and an argon inlet was charged with 3,3-bis(1H-imidazol-1-yl)propan-1-ol ligand (1.2 eq) in 10 mL of dry DMF. At 0°C NaH (60% in mineral oil; 3 eq) was slowly added and the mixture was kept under stirring for 20 min and for additional 30 min at 28 °C. Then the resin SP02-Cl was added (0.8 g) and the mixture was kept under stirring at 60 °C for 24 h. The resulting material was washed with water and acetone and dried under vacuum. The polymeric material was placed in presence of of 5 mL of MeI and kept under stirring at 90°C. After 20 h the resulting POLITAG-I was filtered and washed with methanol and acetone and dried under vacuum. Loading of bis-imidazolium units supported on polymer were determined by elemental analysis (**POLITAG-L-I**: C: 61.360; N: 3.09; H: 6.366; 0.55 mmol/g ligand loading, 1.1 mmol/g $I^-$ loading; POLITAG-M-I: C: 49.32; N: 5.63; H: 5.837; 1.01 mmol/g ligand loading, 2.02 mmol/g $I^-$ loading)

*Synthesis of **POLITAGs-F*****.**

POLITAG-I was charged onto a chromatographic column and an aqueous solution of KF (2M) was passed through the material until no silver iodide precipitate from the eluted solution. After the completion of the ionic exchange the resin was washed with acetone and dried under vacuum. Loading of bis-imidazolium units supported on polymer were determined by elemental analysis (**POLITAG-L-F**: C: 71.88; N: 3.48; H: 9.878; 0.62 mmol/g ligand loading, 1.24 mmol/g $F^-$ loading; **POLITAG-M-F**: C: 68.64; N: 7.43; H: 8.353; 1.33 mmol/g of ligand, 2.66 mmol/g of $F^-$).

*Typical procedure for β-azidation reaction (1 mmol scale)*

In a screw capped vial equipped with a magnetic stirrer (3E)-hept-3-en-2-one (**1a**) (134 µL, 1 mmol), **POLITAG-M-F** (0.0188 g, 0.05 mmol, 2.66 mmol/g of fluoride), azidotrimethylsilane (138 µL, 1.05 mmol) and azeotrope $CH_3CN$:$H_2O$ (200 µL, 5 M) were consecutively added and the resulting mixture was left under vigorous stirring at 60 °C. After 2.5 h, 3 mL of azeotrope $CH_3CN$:$H_2O$ was added, the

catalyst was recovered by filtration and the organic solvent distilled and recovered at 96 % to give pure 4-azidoheptan-2-one (**2a**) as an oil (97 % yield, 150 mg).

### *Recycle of* ***POLITAG-M-F***

The crude reaction mixture was filtered off from the vial and the **POLITAG-M-F** was washed for 3 times with 1 mL of azeotrope $CH_3CN:H_2O$, each time the azeotrope used was filtered off and collected. The **POLITAG-M-F** was dried at 100°C under vacuum for 3 h and reused without significant mass losses.

### *β-azidation reaction on 10 mmol.*

In a screw capped vial equipped with a magnetic stirrer (3E)-hept-3-en-2-one (**1a**) (1.34 mL, 10 mmol), **POLITAG-M-F** (0.188 g, 0.5 mmol, 2.66 mmol/g of fluoride), azidotrimethylsilane (1.37 mL, 10.2 mmol) and azeotrope $CH_3CN:H_2O$ (2 mL, 5 M) were consecutively added and the resulting mixture was left under vigorous stirring at 60 °C. After 2.5 h, 3 mL of azeotrope $CH_3CN:H_2O$ was added, the catalyst was recovered by filtration and the organic solvent distilled and recovered at 96 % to give pure 4-azidoheptan-2-one (**2a**) as an oil (99 % yield, 1.53 g).

***Caution!*** $TMSN_3$ is a flammable liquid with GHS Category 2. Keep away from heat/sparks/open flames/hot surfaces. - No smoking. Wear protective gloves/protective clothing/eye protection/face protection. Avoid breathing dust/fume/gas/mist/vapors/spray. The procedure should be carried out in a fume hood.

### *Large-scale flow procedure for the β-azidation reaction.*

(3E)-hept-3-en-2-one (**1a**) (6.6 mL, 50 mmol), azeotrope $CH_3CN:H_2O$ (10 mL, 5 M) and azidotrimethylsilane (6.9 mL, 1.05 eq) were pumped into a PTFE reactor packed with **POLITAG-M-F** (947 mg) thermostat at 60 °C with a flow rate of 0.1 mL/min and a BPR of 10 atm. After a residence time of 40 min the product was collected and the line washed with 10 mL of azeotrope $CH_3CN:H_2O$. The azeotrope was distilled and the desired product (**2a**) was obtained as an oil (7.66 g, yield 99%).

**References:**


1. Lauzon, S.; Keipour, H.; Gandon, V.; Ollevier, T. Asymmetric FeII-Catalyzed Thia-Michael Addition Reaction to α,β-Unsaturated Oxazolidin-2-one Derivatives. *Org. Lett.* **2017**, *19*, 23, 6324–6327
2. Kozell, V.; Rahmi, F.; Piermattti, O.; Lanari, D.; Vaccaro, L. A stereoselective organic base-catalyzed protocol for hydroamination of alkynes under solvent-free conditions. *Mol. Cat.* **2018**, *455*, 188-191
3. Marrocchi, A.; Adriaensens, P.; Bartollini, E.; Barkakaty, B.; Carleer, R.; Chen, J.; Vaccaro, L. Novel cross-linked polystyrenes with large space network as tailor-made catalyst supports for sustainable media. *Eur. Polym. J*. **2015**, *73*, 391–401.

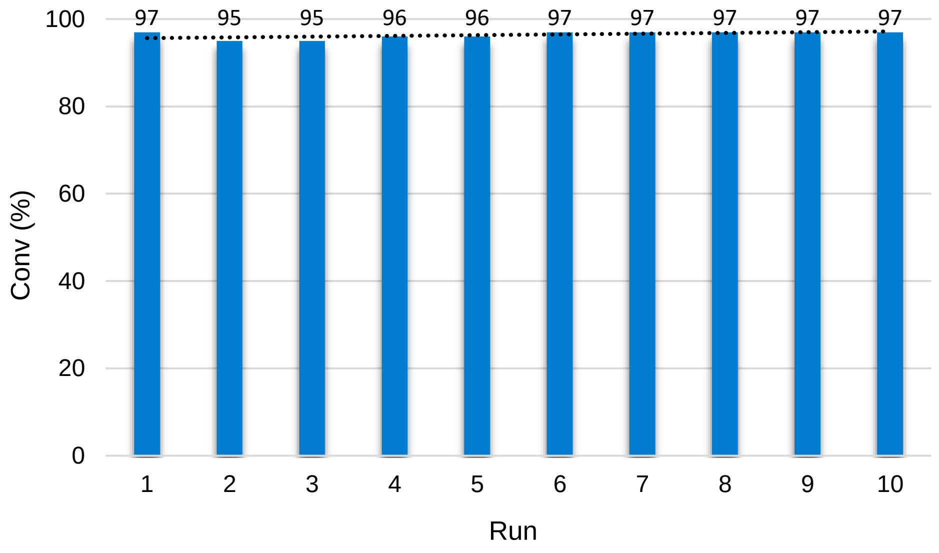


**Figure S1.** Recycle of **POLITAG-M-F** for ten consecutive runs

**Table S1.** Optimization of reaction condition in flow.[a]

| entry | Reactor length (m) | ϕ | POLITAG-M-F(mg) | Residence time (min) | Conc. (M) | Conv (%)[b] |
|---|---|---|---|---|---|---|
| **1** | 0.25 | 4.5 mm | 190 | 10.7 | 5 | 20 |
| **2** | 0.25 | 4.5 mm | 380 | 10.7 | 5 | 22 |
| **3** | 0.50 | 4.5 mm | 758 | 21.4 | 5 | 32 |
| **4** | 0.75 | 4.5 mm | 95 | 32 | 5 | 42 |
| **5** | 1.6 | 1/8” | 474 | 20 | 1 | 35 |
| **6** | 1.6 | 1/8” | 474 | 20 | 5 | 56 |
| **7** | 4.5 | 1/8” | 2686 | 58 | 1 | 99 |
| **8** | 3.2 | 1/8” | 947 | 40 | 5 | 99 |

[a]Reaction condition: **1a** (5mmol), $TMSN_3$ (1.05), $CH_3CN$:$H_2O_{Az.}$, 60 °C. [b]Conversion determined by GC analysis. The remained material is unreacted **1a**.

## 3. E-Factor Calculation and green assessment analysis

***This work*** 1 mmol scale:

**Reactants:**

**1a**: 0.112 g

$TMSN_3$: 0.12 g

$H_2O$: 0.01802 g

**Reaction Medium:**

Azeotrope $CH_3CN/H_2O$: 0.1642 g

**Catalysts:**

**POLITAG-M-F**: 0.0188 g

**Workup Materials:**

Azeotrope $CH_3CN/H_2O$: 2.463 g

**Purification** → not performed

**Recycling-Recovery of Materials:**

**POLITAG-M-F**: 0.0188 g

Azeotrope $CH_3CN/H_2O$ (reaction medium): 0.159 g

Azeotrope $CH_3CN/H_2O$ (work-up): 2.374 g

**2a**: 0.15 g (yield 97%)

E-factor: $((0.250\ g)_{reactants} + (0.1642\ g)_{reaction\ medium} + (0.0188\ g)_{catalyst} + (2.463\ g)_{work\text{-}up} – (2.552\ g)_{mass\ recovered} – (0.150\ g)_{product}) / (0.150\ g)_{product}$ = **1.296**

*J. Am. Chem. Soc.* **2005,** *127*, 1313–1317:

**Reactants:**

**1a**: 0.112 g

$NaN_3$: 0.13 g

HCl: 0.072916 g

**Reaction Medium:**

methylcyclohexane: 3.850 g

**Catalyst:** 0.028 g

**Workup Materials:**

NaOH (1M): 20.8 g

diethyl ether: 10.695 g

**Purification** → not considered in the calculation

**2a**: 0.136 g (yield 88%)

E-factor: $((0.315\ g)_{reactants} + (3.850\ g)_{reaction\ medium} + (0.028\ g)_{catalyst} + (31.495\ g)_{work\text{-}up} – (0.136\ g)_{product}) / (0.136\ g)_{product}$ = **261.411**

*J. Org. Chem*. **2006**, *71*, 9536–9539:

**Reactants:**

**1a**: 0.116 g

$TMSN_3$: 0.131 g

$H_2O$: 0.01802 g

**Reaction Medium:**

SolFC

**Catalysts:**

**Amberlyte IRA900-F**: 0.038 g

**Workup Materials:**

EtOAc: 0.902 g

**Purification** → not performed

**Recycling-Recovery of Materials:**

**Amberlyte IRA900-F**: 0.038 g

**2a**: 0.140 g (yield 87%)

E-factor: $((0.265\ g)_{reactants} + (0.038\ g)_{catalyst} + (0.902\ g)_{work\text{-}up} – (0.038\ g)_{mass\ recovered} – (0.140\ g)_{product}) / (0.140\ g)_{product}$ = **7.336**

*Adv. Synth. Catal*. **2012**, *354*, 908–916:

**Reactants:**

**1a**: 0.056 g

TMS$N_3$: 0.0876 g

$H_2O$: 0.00901 g

**Reaction Medium:**

$H_2O$: 0.241 g

**Catalysts:**

**PS-DABCO-$F_2$**: 0.014 g

**Workup Materials:**

EtOAc: 0.451 g

**Purification** → not performed

**Recycling-Recovery of Materials:**

**PS-DABCO-$F_2$**: 0.014 g

$H_2O$: 0.239 g

**2a**: 0.07 g (yield 90%)

E-factor: $((0.153\ g)_{reactants} + (0.241\ g)_{reaction\ medium} + (0.014\ g)_{catalyst} + (0.451\ g)_{work\text{-}up} – (0.253\ g)_{mass\ recovered} – (0.070\ g)_{product}) / (0.070\ g)_{product}$ = **7.649**

***This work*** 10 mmol scale:

**Reactants:**

**1a**: 1.12 g

$TMSN_3$: 1.166 g

$H_2O$: 0.1802 g

**Reaction Medium:**

Azeotrope $CH_3CN/H_2O$: 1.642 g

**Catalysts:**

**POLITAG-M-F**: 0.188 g

**Workup Materials:**

Azeotrope $CH_3CN/H_2O$: 2.463 g

**Purification** → not performed

**Recycling-Recovery of Materials:**

**POLITAG-M-F**: 0.188 g

Azeotrope $CH_3CN/H_2O$ (reaction medium): 1.576 g

Azeotrope $CH_3CN/H_2O$ (work-up): 2.374 g

**2a**: 1.53 g (yield 99%)

E-factor: $((2.466\text{ g})_{\text{reactants}} + (1.642\text{ g})_{\text{reaction medium}} + (0.188\text{ g})_{\text{catalyst}} + (2.463\text{ g})_{\text{work-up}} - (4.2013\text{ g})_{\text{mass recovered}} - (1.530\text{ g})_{\text{product}}) / (1.530\text{ g})_{\text{product}}$ = **0.672**

**Table S2.** Comparison of different green metrics for the synthesis of products **2a**, **2j** and **2k** via β-azidation reaction of α,β-unsaturated carbonyl compounds.

| product | ref | AE | RME | MRP | 1/SF | Rxn Yield | VMR |
|---|---|---|---|---|---|---|---|
| **2a** | *This work* | 0.632 | 0.436 | 0.726 | 0.980 | 0.968 | 0.776 |
| | 15 | 0.726 | 0.004 | 0.009 | 0.677 | 0.878 | 0.593 |
| | 37 | 0.632 | 0.120 | 0.227 | 0.958 | 0.872 | 0.655 |
| | 35 | 0.632 | 0.116 | 0.252 | 0.803 | 0.903 | 0.623 |
| **2j** | *This work* | 0.685 | 0.475 | 0.740 | 0.984 | 0.953 | 0.790 |
| | 20 | 0.530 | 0.001 | 0.003 | 0.534 | 0.970 | 0.545 |
| | 21 | 0.530 | 0.053 | 0.193 | 0.534 | 0.970 | 0.556 |
| **2k** | *This work* | 0.687 | 0.460 | 0.742 | 0.984 | 0.928 | 0.781 |
| | 36 | 0.687 | 0.049 | 0.090 | 0.797 | 0.994 | 0.649 |
| | 22 | 0.600 | 0.031 | 0.129 | 0.418 | 0.950 | 0.539 |
| | 20 | 0.532 | 0.001 | 0.003 | 0.536 | 0.850 | 0.508 |
| | 21 | 0.532 | 0.047 | 0.193 | 0.536 | 0.850 | 0.516 |

## 4. Benign Index and Safety Hazard Index data

The safety/hazard analyses have been conducted in accordance with the methods, calculations and equation reported by J. Andraos in *Org. Process Res. Dev*. **2013**, *17*, 175−192.

The benign index analyses, definitions, and equation for multicompartment method (MCM) have been used in accordance with the data and equations reported by J. Andraos in *Org. Process Res. Dev*. **2012**, *16*, 1482−1506.

Log Kow data are reported by J. Andraos in *ACS Sustainable Chem. Eng*. **2018**, *6*, 3206–3214 and by J. Andraos and L. Vaccaro in *Green Chem*., **2015**, *17*, 913-925.
LD50 (oral and dermal), LC50, OEL, Flash point and risk phrases references and sources: MSDS files for each compound, Registry of Toxic Effects of Chemical Substances (RTECS) and NIOSH POCKET GUIDE TO CHEMICAL HAZARDS, National Institute for Occupational Safety and Health, September 2007, Publication No. 2005-149 (http://www.cdc.gov/niosh).

**Table S3.** Substance parameters.

| compound | LD50 oral (mg/Kg) | LD50 dermal (mg/Kg) | LC50 ($g/m^3$, 4h) | Log Kow | OEL ($mmol/m^3$) | SD (mg) |
|---|---|---|---|---|---|---|
| 3-hepten-2-one | - | 5000 | - | 1.8 | - | 65.7 |
| $TMSN_3$ | 100 | 300 | 3 | -4.17 | - | 45.5 |
| $NaN_3$ | 27 | 20 | 27 | $-1\cdot10^{100}$ | $4.46\cdot10^{-3}$ | 22.1 |
| HCl | - | >5010 | 2.3 | 1.1 | 7 | 2184.5 |
| TMSOH | 2800 | 2800 | 3151 | 1.62 | - | 1101.25 |
| NaCl | 3000 | 10000 | 42000 | $-1\cdot10^{100}$ | - | - |

**Table S4.** Safety Hazard Index (SHI) parameters.

| compound | CLP | CGP | FP | XVP | OELP | SDP | RPP | Ω(SHI) |
|---|---|---|---|---|---|---|---|---|
| 3-hepten-2-one | 0.8036 | - | 0.7015 | - | - | 0.9877 | 1.4087 | 3.5479 |
| $TMSN_3$ | 7.5 | 0.1733 | 0.8172 | - | - | 0.6840 | 2.5324 | 11.707 |
| $NaN_3$ | 112.5 | 0.0193 | - | - | 913.4917 | 0.3322 | 1.2822 | 1027.63 |
| HCl | 0.4491 | 0.2261 | - | - | 0.5823 | 32.8398 | 1 | 35.097 |
| TMSOH | 0.8036 | 0.000165 | 0.8231 | 3.4 | - | 16.555 | 0.8785 | 22.46 |
| NaCl | 0.225 | $1.24\cdot10^{-5}$ | - | - | - | - | - | 0.225 |

**Table S5.** Benign Index (BI) parameters.

| compound | GWP | INHTP | INGTP | BCP | Ω(BI) |
|---|---|---|---|---|---|
| 3-hepten-2-one | 2.75 | - | - | 0.196 | 2.95 |
| $TMSN_3$ | 1.15 | $4.74\cdot10^{-5}$ | $6.78\cdot10^{2}$ | $5.702\cdot10^{-6}$ | 679 |
| $NaN_3$ | 0 | $5.26\cdot10^{-96}$ | $4.45\cdot10^{3}$ | 0 | 4450 |
| HCl | 0 | 27.9 | - | 0.058 | 48.5 |
| TMSOH | 1.47 | $7.99\cdot10^{-3}$ | 15 | 0.143 | 16.6 |
| NaCl | 0 | $3.77\cdot10^{-99}$ | 44.6 | 0 | 44.6 |

**Table S6.** Fractional weight of the input materials.

$$f_x = \frac{weight_x}{\sum_x weight_x}$$

| reference | $f_{3\text{-hepten-2-one}}$ | $f_{TMSN3}$ | $f_{NaN3}$ | $f_{HCl}$ |
|---|---|---|---|---|
| *This work* | 0.448 | 0.48 | - | - |
| *J. Am. Chem. Soc.* **2005**, ***127***, 1313–1317 | 0.3556 | - | 0.4127 | 0.2317 |

**Table S7.** Fractional weight of the waste materials.

| reference | $f_{TMSN3}$ | $f_{NaN3}$ | $f_{HCl}$ | $f_{TMSOH}$ | $f_{NaCl}$ |
|---|---|---|---|---|---|
| *This work* | 0.0526 | - | - | 0.9474 | - |
| *J. Am. Chem. Soc.* **2005**, *127*, 1313–1317 | - | 0.4064 | 0.228 | - | 0.3650 |

**Table S8.** BI and SHI calculation over fractional weight

| reference | $SHI_{(input)}$ | $BI_{(input)}$ | $BI_{(waste)}$ |
|---|---|---|---|
| *This work* | 0.52744 | 0.5201 | 0.9260 |
| *J. Am. Chem. Soc*. **2005**, *127*, 1313–1317 | 0.59345 | 0.5892 | 0.5959 |

*Reaction media, solvents used in the work-up and catalysts were excluded by the calculation. SHI = 1 ideal situation. BI = 1 ideal situation*

**Abbreviations:**

BCP = Bioconcentration Potential
CLP = Corrosiveness potential as a liquid/solid
CGP = Corrosiveness potential as a gas
FP = Flammability potential
GWP = Global Warming Potential
INHTP = Inhalation Toxicity Potential
INGTP = Ingestion Toxicity Potential
RPP = Risk Phrase Potential
OELP = Occupational Exposure Limit Potential
SDP = Skin Dose Potential
XVP = Explosive Vapor Potential

## 5. Characterization data

| | |
|---|---|
| **Cchem. Name** | **4-azidoheptan-2-one (2a)** |
| **Lit. Ref.** | *J. Org. Chem.,* **2006**, 71, 9536-9539 |

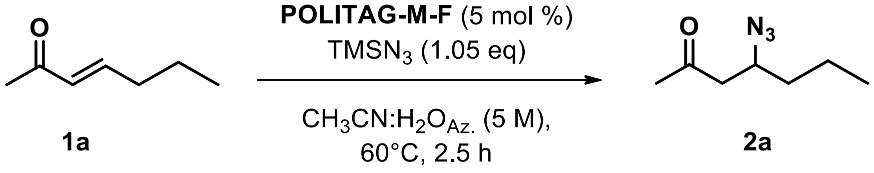


**1a** **2a**

M.W.: 155.20 g/mol

**METHOD:**
In a screw capped vial equipped with a magnetic stirrer (*E*)-hept-3-en-2-one (**1a**) (134 μL, 1 mmol, 98 %), **POLITAG-M-F** (0.0188 g, 5 mol%, 2.66 mmol/g of fluoride), azidotrimethylsilane (138 μL, 1.05 mmol) and azeotrope $CH_3CN:H_2O$ (200 μL, 5 M) were consecutively added and the resulting mixture was left under vigorous stirring at 60 °C. After 2.5 h, 3 mL of azeotrope $CH_3CN:H_2O$ was added, the catalyst was recovered by filtration and the organic solvent distilled and recovered at 96 % to give pure 4-azidoheptan-2-one (**2a**) as an oil (97 % yield, 150 mg).

E-Factor = (112 mg (**1a**) + 120 mg ($TMSN_3$) + 94 mg (azeotrope not recovered) – 150 mg (**2a**))/ 150 mg (**2a**) = **1.3**

| **Mol Formula** | $C_7H_{13}N_3O$ | **m.p.** | oil |
|---|---|---|---|

| **$^1$H NMR 400 MHz $CDCl_3$** | **δ value** | **No. H** | **Mult.** | **j value/Hz** |
|---|---|---|---|---|
| | 3.87-3.82 | 1 | *m* | |
| | 2.67 | 1 | *dd* | 17.2, 8.3 |
| | 2.54 | 1 | *dd* | 17.2, 4.6 |
| | 2.18 | 3 | *s* | |
| | 1.53-1.37 | 4 | *m* | |
| | 0.98-0.92 | 3 | *m* | |

**$^{13}$C NMR (100.6 MHz, $CDCl_3$) δ :** 206, 58, 48.2, 36.7, 30.7, 19.4, 13.9.

**GC-EIMS (m/z, %):** 155 (M+, < 1); 99 (15); 58 (25); 43 (100).

| | |
|---|---|
| **Chem. Name** | **4-azidononan-2-one** |
| **Lit. Ref.** | *J. Org. Chem.,* **2006**, 71, 9536-9539 |

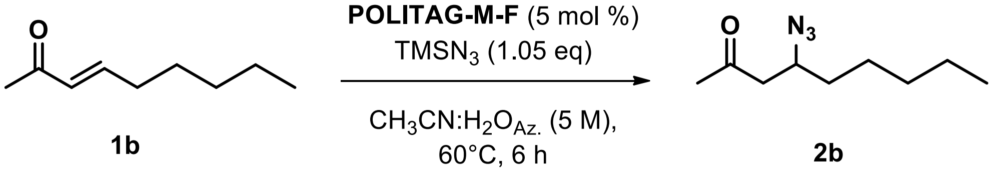


**1b** **2b**

M.W.: 183.26 g/mol

**METHOD:**
In a screw capped vial equipped with a magnetic stirrer (E)-non-3-en-2-one (**1b**) (169 μL, 1 mmol, 95 %), **POLITAG-M-**F (0.0188 g, 5 mol%, 2.66 mmol/g of fluoride), azidotrimethylsilane (138 μL, 1.05 mmol) and azeotrope $CH_3CN:H_2O$ (200 μL, 5 M) were consecutively added and the resulting mixture was left under vigorous stirring at 60 °C. After 6 h, 3 mL of azeotrope $CH_3CN:H_2O$ was added, the catalyst was recovered by filtration and the organic solvent distilled and recovered at 96 % to give pure 4-azidononan-2-one (**2b**) as an oil (97 % yield, 177 mg).

E-Factor = (140 mg (**1b**) + 120 mg ($TMSN_3$) + 94 mg (azeotrope not recovered) – 177 mg (**2b**))/ 177 mg (**2b**) = 1.0

| **$^1$H NMR 400 MHz $CDCl_3$** | **δ value** | **No. H** | **Mult.** | **j value/Hz** |
|---|---|---|---|---|
| | 3.88-3.80 | 1 | *m* | |
| | 2.67 | 1 | *dd* | 17.2, 8.3 |
| | 2.54 | 1 | *dd* | 17.2, 4.6 |
| | 2.19 | 3 | *s* | |
| | 1.52-1.47 | 2 | *m* | |
| | 1.32-1.28 | 6 | *m* | |
| | 0.92-0.87 | 3 | *m* | |

**$^{13}$C NMR (100.6 MHz, $CDCl_3$) δ :** 205.9, 58.1, 48, 34.4, 31.4, 30.6, 25.6, 22.5, 13.9.

**GC-EIMS (m/z, %):** 183 (M+, < 1); 99 (20); 57 (100).

| Chem. Name | **1-azidopentan-3-one** |
|---|---|
| **Lit. Ref.** | *J. Org. Chem.,* **2006**, 71, 9536-9539 |

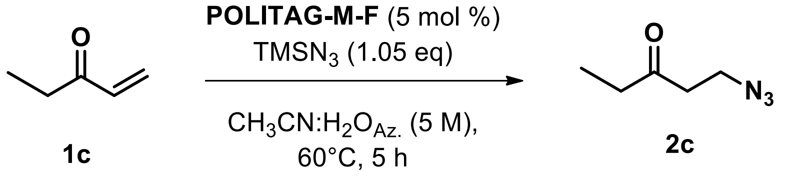


M.W.: 127.15 g/mol

**METHOD:**
In a screw capped vial equipped with a magnetic stirrer pent-1-en-3-one (**1c**) (103 µL, 1 mmol, 97 %), **POLITAG-M-F** (0.0188 g, 5 mol%, 2.66 mmol/g of fluoride), azidotrimethylsilane (138 µL, 1.05 mmol) and azeotrope $CH_3CN:H_2O$ (200 µL, 5 M) were consecutively added and the resulting mixture was left under vigorous stirring at 60 °C. After 5 h, 3 mL of azeotrope $CH_3CN:H_2O$ was added, the catalyst was recovered by filtration and the organic solvent distilled and recovered at 96 % to give pure 1-azidopentan-3-one (**2c**) as an oil (83 % yield, 106 mg).

E-Factor = (84 mg (**1c**) + 120 mg ($TMSN_3$) + 94 mg (azeotrope not recovered) – 106 mg (**2c**))/ 106 mg (**2c**) = 1.8

| **Mol Formula** | | $C_5H_9N_3O$ | | **m.p.** | Oil |
|---|---|---|---|---|---|
| **$^1$H NMR 400 MHz $CDCl_3$** | **δ value** | **No. H** | **Mult.** | **j value/Hz** | |
| | 3.55 | 2 | *t* | 6.4 | |
| | 2.67 | 2 | *t* | 6.4 | |
| | 2.47 | 2 | *q* | 7.3 | |
| | 1.08 | 3 | *t* | 7.3 | |

**$^{13}$C NMR (100.6 MHz, $CDCl_3$) δ :** 208.4, 45.8, 41, 36.3, 7.6

**GC-EIMS (m/z, %):** 127 (M+, < 1); 72 (77); 57 (100).

| Chem. Name | 4-azidobutan-2-one |
|---|---|
| Lit. Ref. | |

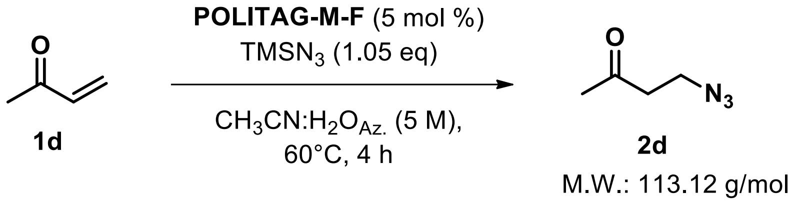


**METHOD:**

In a screw capped vial equipped with a magnetic stirrer but-3-en-2-one (**1d**) (86 µL, 1 mmol, 99 %), **POLITAG-M-F** (0.0188 g, 5 mol%, 2.66 mmol/g of fluoride), azidotrimethylsilane (138 µL, 1.05 mmol) and azeotrope $CH_3CN:H_2O$ (200 µL, 5 M) were consecutively added and the resulting mixture was left under vigorous stirring at 60 °C. After 4 h, 3 mL of azeotrope $CH_3CN:H_2O$ was added, the catalyst was recovered by filtration and the organic solvent distilled and recovered at 96 % to give pure 4-azidobutan-2-one (**2d**) as an oil (84 % yield, 95 mg).

E-Factor **2d** = (70 mg (**1d**) + 120 mg ($TMSN_3$) + 94 mg (azeotrope not recovered) – 95 mg (**2d**))/ 95 mg (**2d**) = 2.0

| **Mol Formula** | | $C_4H_7N_3O$ | | **m.p.** | oil |
|---|---|---|---|---|---|
| **$^1$H NMR 400 MHz $CDCl_3$** | **δ value** | **No. H** | **Mult.** | **j value/Hz** | |
| | 3.54 | 2 | *t* | 6.4 | |
| | 2.70 | 2 | *t* | 6.4 | |
| | 2.19 | 3 | *s* | | |

**$^{13}$C NMR (100.6 MHz, $CDCl_3$) δ :** 205.8, 45.8, 42.5, 30.4.

**GC-EIMS (m/z, %):** 113 (M+, < 1); 58 (100).

| **Chem. Name** | **3-azido-1-(2,6,6-trimethylcyclohex-1-en-1-yl)butan-1-one** |
|---|---|
| **Lit. Ref.** | *J. Org. Chem.,* **2006**, 71, 9536-9539 |

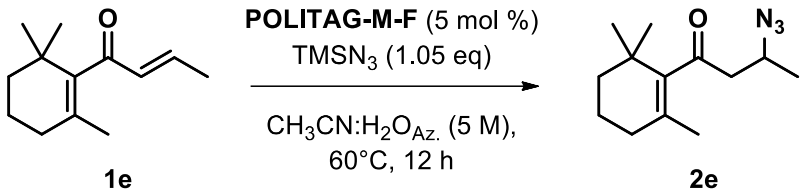


M.W.: 235.33 g/mol

**METHOD:**
In a screw capped vial equipped with a magnetic stirrer β-Damascone (**1e**) (229 μL, 1 mmol, 90 %), **POLITAG-M-F** (0.0188 g, 5 mol%, 2.66 mmol/g of fluoride), azidotrimethylsilane (138 μL, 1.05 mmol) and azeotrope $CH_3CN$:$H_2O$ (200 μL, 5 M) were consecutively added and the resulting mixture was left under vigorous stirring at 60 °C. After 12 h, 3 mL of azeotrope $CH_3CN$:$H_2O$ was added, the catalyst was recovered by filtration and the organic solvent distilled and recovered at 96 % to give pure 3-azido-1-(2,6,6-trimethylcyclohex-1-en-1-yl)butan-1-one (**2e**) as an oil (96 % yield, 226 mg).

E-Factor = (192 mg (**1e**) + 120 mg ($TMSN_3$) + 94 mg (azeotrope not recovered) – 226 mg (**2e**))/ 226 mg (**2e**) = 0.8

| **Mol Formula** | | $C_{13}H_{21}N_3O$ | | **m.p.** | oil |
|---|---|---|---|---|---|
| **$^1$H NMR 400 MHz $CDCl_3$** | **δ value** | **No. H** | **Mult.** | **j value/Hz** | |
| | 4.16-4.05 | 1 | *m* | | |
| | 2.83 | 1 | *dd* | 18.6, 7.5 | |
| | 2.58 | 1 | *dd* | 18.5, 5.4 | |
| | 1.95 | 2 | *t* | 6.4 | |
| | 1.68-1.62 | 2 | *m* | | |
| | 1.58 | 3 | *s* | | |
| | 1.45-1.42 | 2 | *m* | | |
| | 1.31 | 3 | *d* | 6.6 | |
| | 1.06 | 6 | *d* | 11.3 | |

**$^{13}$C NMR (100.6 MHz, $CDCl_3$) δ :** 208.4, 142.7, 130.1, 53.1, 51.9, 39, 31.4, 28.9, 28.7, 20.9, 19.8, 18.9.

**GC-EIMS (m/z, %):** 235 (M +, < 1); 192 (59); 165 (11); 164 (32); 152 (10); 151 (86); 138 (22); 137(17); 136 (11); 135 (19); 124 (13); 123 (100); 109 (12); 107 (24); 105 (10); 95 (26); 93 (20); 91 (28); 81 (89); 79 (37); 77 (24); 69 (18); 67 (25); 65 (14); 57 (12); 56 (10); 55 (23); 53 (17).

| Chem. Name | **(5R)-2-(2-azidopropan-2-yl)-5-methylcyclohexanone** |
|---|---|
| **Lit. Ref.** | *J. Org. Chem.,* **2006**, 71, 9536-9539 |

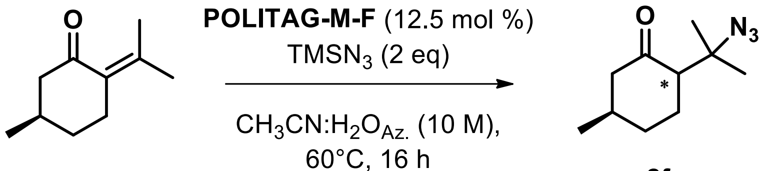


**1f** **2f**

M.W.: 195.27 g/mol

**METHOD:**

In a screw capped vial equipped with a magnetic stirrer (R)-(+)-Pulegone (**1f**) (166 μL, 1 mmol, 95 %), **POLITAG-M-F** (0.0470g, 12.5 mol%, 2.66 mmol/g of fluoride), azidotrimethylsilane (268 μL, 2 mmol) and azeotrope $CH_3CN:H_2O$ (100 μL, 10 M) were consecutively added and the resulting mixture was left under vigorous stirring at 60 °C. After 16 h, 3 mL of azeotrope $CH_3CN:H_2O$ was added, the catalyst was recovered by filtration and the organic solvent distilled and recovered at 96 % to give a 60/40 mixture of two diastereoisomers of (5R)-2-(2-azidopropan-2-yl)-5-methylcyclohexanone (**2f**) as an oil (96 % yield, 187 mg).

E-Factor = (152 mg (**1f**) + 230 mg ($TMSN_3$) + 94 mg (azeotrope not recovered) – 187 mg (**2f**))/ 187 mg (**2f**) = 1.6

| **Mol Formula** | | $C_{10}H_{17}N_3O$ | | **m.p.** | oil |
|---|---|---|---|---|---|
| **$^1$H NMR 400 MHz $CDCl_3$** | **δ value** | **No. H** | **Mult.** | **j value/Hz** | |
| | 2.54-2.1 | 6 | *m* | | |
| | 2.1-1.74 | 10 | *m* | | |
| | 1.42 | 6 | *s* | | |
| | 1.34 | 6 | *s* | | |
| | 1-02 | 3 | *d* | 6.3 | |
| | 0.94 | 3 | *d* | 7.1 | |

**$^{13}$C NMR (100.6 MHz, $CDCl_3$) δ :** 210.8, 209.9, 62.5, 58.3, 58.1, 52, 50, 36.3, 34.3, 32.6, 30.9, 28.7, 25.3, 25.1, 24.7, 23, 22.4, 22.3, 19.3

**GC-EIMS (m/z, %):** 195 (M+ < 1); 109 (20); 69 ( 31); 56 (100).

| | |
|---|---|
| **Chem. Name** | **3-azidocyclopentanone** |
| **Lit. Ref.** | *J. Org. Chem.,* **2006**, 71, 9536-9539 |

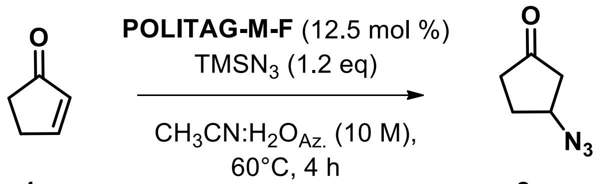


**1g** **2g**

M.W.: 125.13 g/mol

**METHOD:**

In a screw capped vial equipped with a magnetic stirrer 3-azidocyclopentanone (**1g**) (134 μL, 1 mmol, 98 %), **POLITAG-M-F** (0.0470 g, 12.5 mol%, 2.66 mmol/g of fluoride), azidotrimethylsilane (161 μL, 1.2 mmol) and azeotrope $CH_3CN$:$H_2O$ (100 μL, 10 M) were consecutively added and the resulting mixture was left under vigorous stirring at 60 °C. After 4 h, 3 mL of azeotrope $CH_3CN$:$H_2O$ was added, the catalyst was recovered by filtration and the organic solvent distilled and recovered at 96 % to give pure 3-azidocyclopentanone (**2g**) as an oil (84 % yield, 105 mg).

E-Factor = (82 mg (**1g**) + 138 mg ($TMSN_3$) + 94 mg (azeotrope not recovered) –105 mg (**2g**))/ 105 mg (**2g**) = 2

| **Mol Formula** | | $C_5H_7N_3O$ | | **m.p.** | oil |
|---|---|---|---|---|---|
| **$^1$H NMR 400 MHz $CDCl_3$** | **δ value** | **No. H** | **Mult.** | **j value/Hz** | |
| | 4.31-4.32 | 1 | *m* | | |
| | 2.38-2.50 | 1 | *m* | | |
| | 2.29-2.38 | 2 | *m* | | |
| | 2.21-2.27 | 2 | *m* | | |
| | 2.12-2.16 | 1 | *m* | | |

**$^{13}$C NMR (100.6 MHz, $CDCl_3$) δ :** 215, 59.1, 44.3, 36, 29.1

**GC-EIMS (m/z, %):** 125 (M+, 17); 99 (22); 98 (33); 97 (100); 82 (38); 68 (66); 67 (22); 54 (58); 52 (26); 52 (15); 50 (30).

| Chem. Name | 3-azidocyclohexanone |
|---|---|
| Lit. Ref. | *J. Org. Chem.,* **2006**, 71, 9536-9539 |

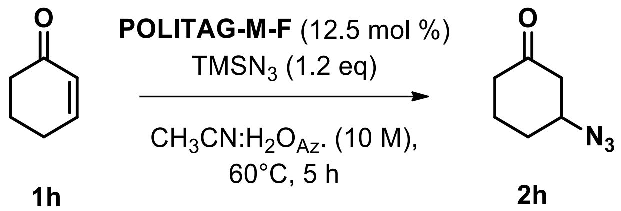


**1h** **2h**

M.W.: 139.16 g/mol

**METHOD:**
In a screw capped vial equipped with a magnetic stirrer cyclohex-2-enone (**1h)** (99 μL, 1 mmol, 95 %), **POLITAG-M-F** (0.0470g, 12.5 mol%, 2.66 mmol/g of fluoride), azidotrimethylsilane (161 μL, 1.2 mmol) and azeotrope $CH_3CN$:$H_2O$ (100 μL, 10 M) were consecutively added and the resulting mixture was left under vigorous stirring at 60 °C. After 5 h, 3 mL of azeotrope $CH_3CN$:$H_2O$ was added, the catalyst was recovered by filtration and the organic solvent distilled and recovered at 96 % to give pure 3-azidocyclohexanone (**2h**) as an oil (92 % yield, 128 mg).

E-Factor = (96 mg (**1h**) + 138 mg ($TMSN_3$) + 94 mg (azeotrope not recovered) – 128 mg (**2h**))/ 128 mg (**2h**) = 1.6

| **Mol Formula** | | $C_6H_9N_3O$ | | **m.p.** | oil |
|---|---|---|---|---|---|
| **$^1$H NMR 400 MHz $CDCl_3$** | **δ value** | **No. H** | **Mult.** | **j value/Hz** | |
| | 3.85-3.91 | 1 | *m* | | |
| | 2.65 | 1 | *dd* | 14.2, 4.6 | |
| | 2.46-2.40 | 1 | *m* | | |
| | 2.32-2.36 | 2 | *m* | | |
| | 2.11-2.05 | 2 | *m* | | |
| | 1.85-1.71 | 2 | *m* | | |

**$^{13}$C NMR (100.6 MHz, $CDCl_3$) δ :** 207.5, 59.5, 46.7, 40.8, 29.8, 21.4

**GC-EIMS (m/z, %):** 139 (M+, 66); 97 (15); 83 (18); 82 (37); 69 (40); 68 (45); 67 (15); 55 (100); 54 (48)

| Chem. Name | **3-azidocycloheptanone** |
|---|---|
| **Lit. Ref.** | *J. Org. Chem.,* **2006**, 71, 9536-9539 |

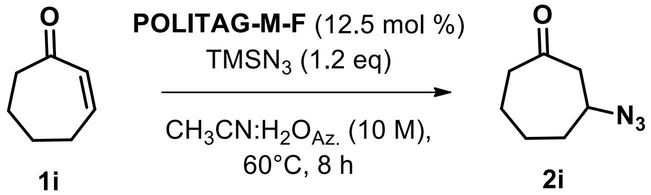


**1i** → **2i**

M.W.: 153.19 g/mol

**METHOD:**

In a screw capped vial equipped with a magnetic stirrer cyclohept-2-enone (**1i**) (134 μL, 1 mmol, 80 %), **POLITAG-M-F** (0.0470 g, 12.5 mol%, 2.66 mmol/g of fluoride), azidotrimethylsilane (161 μL, 1.2 mmol) and azeotrope $CH_3CN:H_2O$ (100 μL, 10 M) were consecutively added and the resulting mixture was left under vigorous stirring at 60 °C. After 8 h, 3 mL of azeotrope $CH_3CN:H_2O$ was added, the catalyst was recovered by filtration and the organic solvent distilled and recovered at 96 % to give pure 4-azidoheptan-2-one (**2i**) as an oil (94 % yield, 144 mg).

E-Factor = (110 mg (**1i**) + 138 mg ($TMSN_3$) + 94 mg (azeotrope not recovered) – 144 mg (**2i**))/ 144 mg (**2i**) = 1.4

| **Mol Formula** | | $C_7H_{11}N_3O$ | | **m.p.** | oil |
|---|---|---|---|---|---|
| **$^1$H NMR 400 MHz $CDCl_3$** | **δ value** | **No. H** | **Mult.** | **j value/Hz** | |
| | 3.82-3.78 | 1 | *m* | | |
| | 2.79-2.76 | 2 | *m* | | |
| | 2.55-2.48 | 3 | *m* | | |
| | 2.00-1.97 | 1 | *m* | | |
| | 1.91-1.88 | 2 | *m* | | |
| | 1.78-1.76 | 2 | *m* | | |
| | 1.64-1.61 | 1 | *m* | | |

**$^{13}$C NMR (100.6 MHz, $CDCl_3$) δ :** 210.4, 57.9, 48.6, 44.2, 35.7, 25.6, 23.7.

**GC-EIMS (m/z, %):** 153 (M+, 14); 109 (25); 81 (11); 69 (29); 56 (100).

| | |
|---|---|
| **Chem. Name** | **1-(3-azidobutanoyl)pyrrolidin-2-one** |
| **Lit. Ref.** | *J. Am. Chem. Soc.*, **2002**, 124, 10, 2134-2136 |

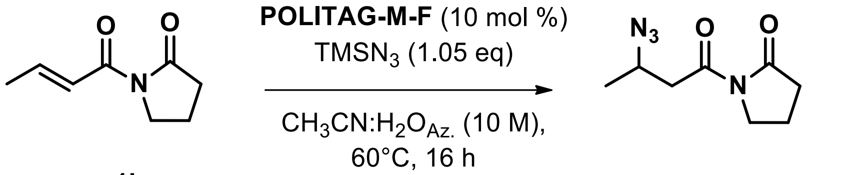


**1j** **2j**

M.W.: 196.21 g/mol

**METHOD:**
In a screw capped vial equipped with a magnetic stirrer (E)-1-(but-2-enoyl)pyrrolidin-2-one (**1j**) (153.18 mg, 1 mmol), **POLITAG-M-F** (0.0375 g, 10 mol%, 2.66 mmol/g of fluoride), azidotrimethylsilane (138 µL, 1.05 mmol) and azeotrope $CH_3CN:H_2O$ (100 µL, 10 M) were consecutively added and the resulting mixture was left under vigorous stirring at 60 °C. After 16 h, 3 mL of azeotrope $CH_3CN:H_2O$ was added, the catalyst was recovered by filtration and the organic solvent distilled and recovered at 96 % to give pure 1-(3-azidobutanoyl)pyrrolidin-2-one (**2j**) as an oil (96 % yield, 187 mg).

E-Factor = (153 mg (**1j**) + 120 mg ($TMSN_3$) + 94 mg (azeotrope not recovered) – 187 mg (**2j**))/ 187 mg (**2j**) = 1.0

| **Mol Formula** | $C_8 H_{12} N_4 O_2$ | **m.p.** | oil |
|---|---|---|---|

| | **δ value** | **No. H** | **Mult.** | **j value/Hz** |
|---|---|---|---|---|
| **$^1$H NMR 400 MHz $CDCl_3$** | 4.12-3.99 | 1 | *m* | |
| | 3.87-3.75 | 2 | *m* | |
| | 3.18 | 2 | *dd* | 17.3-8.4 |
| | 2.96 | 1 | *m* | 17.3-4.6 |
| | 2.59 | 2 | *t* | |
| | 2.10-1.97 | 2 | *m* | |
| | 1.32 | 3 | *d* | 6.6 |

**$^{13}$C NMR (100.6 MHz, $CDCl_3$) δ :** 175.7, 171.2, 53.8, 45.5, 43.4, 33.7, 19.9, 17.3.

| Chem. Name | **3-(3-azidobutanoyl)oxazolidin-2-one** |
|---|---|
| **Lit. Ref.** | *J. Am. Chem. Soc.*, **2002**, 124, 10, 2134-2136 |

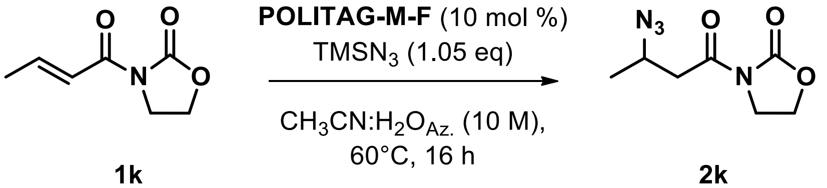


**1k** **2k**

M.W.: 198.18 g/mol

**METHOD:**

In a screw capped vial equipped with a magnetic stirrer (E)-3-(but-2-enoyl)oxazolidin-2-one (**1k**) (155.15 mg, 1 mmol), **POLITAG-M-F** (0.0375 g, 10 mol%, 2.66 mmol/g of fluoride), azidotrimethylsilane (138 μL, 1.05 mmol) and azeotrope $CH_3CN$:$H_2O$ (100 μL, 10 M) were consecutively added and the resulting mixture was left under vigorous stirring at 60 °C. After 16 h, 3 mL of azeotrope $CH_3CN$:$H_2O$ was added, the catalyst was recovered by filtration and the organic solvent distilled and recovered at 96 % to give pure 1-(3-azidobutanoyl)pyrrolidin-2-one (**2k**) as an oil (92 % yield, 182 mg).

E-Factor = (155 mg (**1k**) + 120 mg ($TMSN_3$) + 94 mg (azeotrope not recovered) – 182 mg (**2k**))/ 182 mg (**2k**) = 1.0

| **Mol Formula** | | $C_7 H_{10} N_4 O_3$ | | **m.p.** | Oil |
|---|---|---|---|---|---|
| **$^1$H NMR 400 MHz $CDCl_3$** | **δ value** | **No. H** | **Mult.** | **j value/Hz** | |
| | 4.42 | 2 | *t* | 8.1 | |
| | 4.11-3.95 | 3 | *m* | | |
| | 3.19 | 1 | *dd* | 17.2-8.7 | |
| | 2.96 | 1 | *dd* | 17.2-4.4 | |
| | 1.33 | 3 | *d* | 6.6 | |

**$^{13}$C NMR (100.6 MHz, $CDCl_3$) δ :** 170.4, 153.6, 62.3, 53.8, 42.5, 41.7, 19.8.

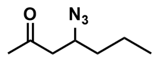


4-azidoheptan-2-one (**2a**)

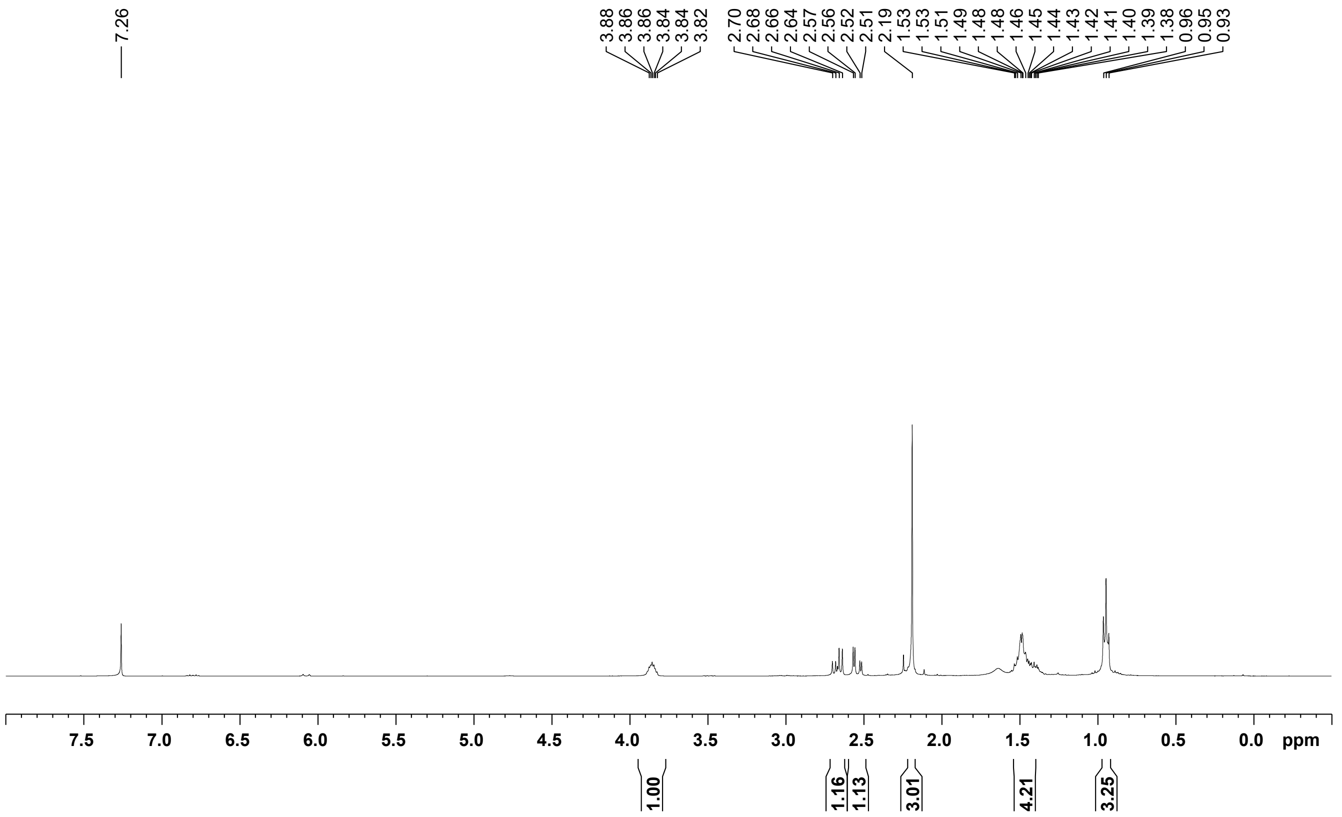


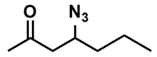


4-azidoheptan-2-one (**2a**)

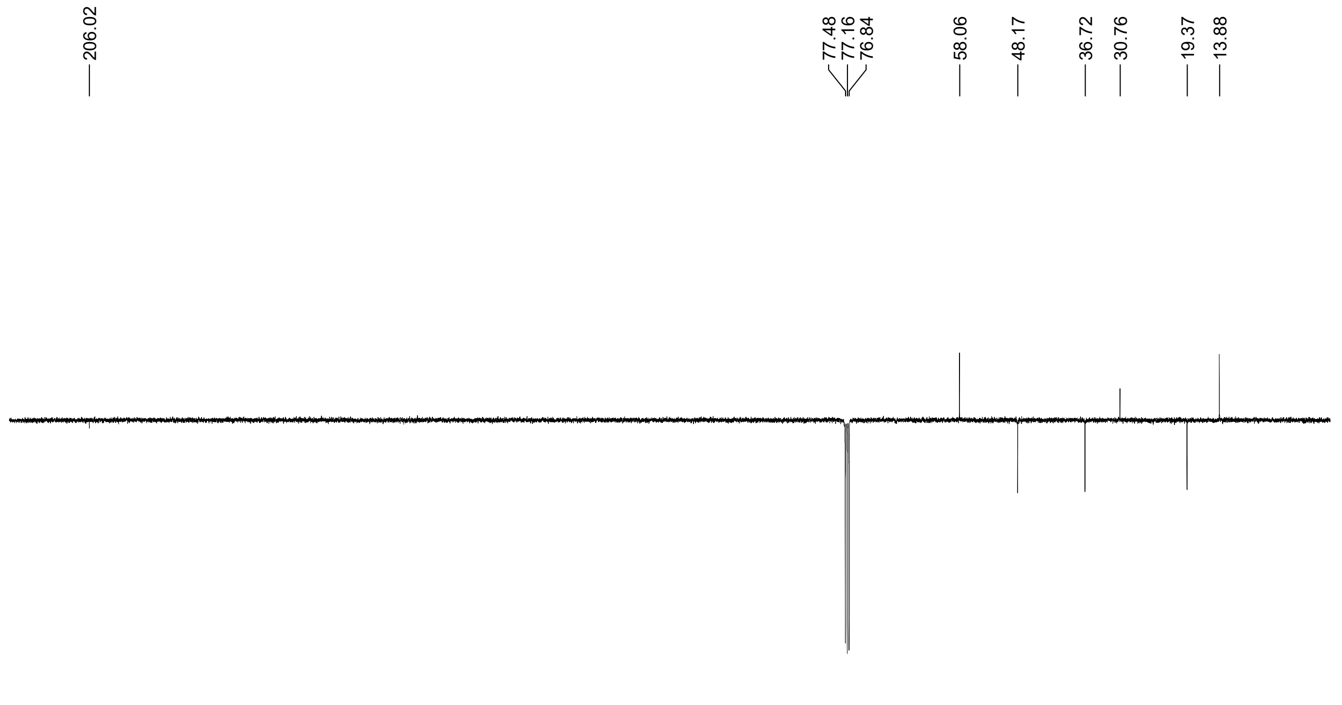

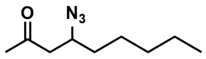


4-azidononan-2-one (**2b**)

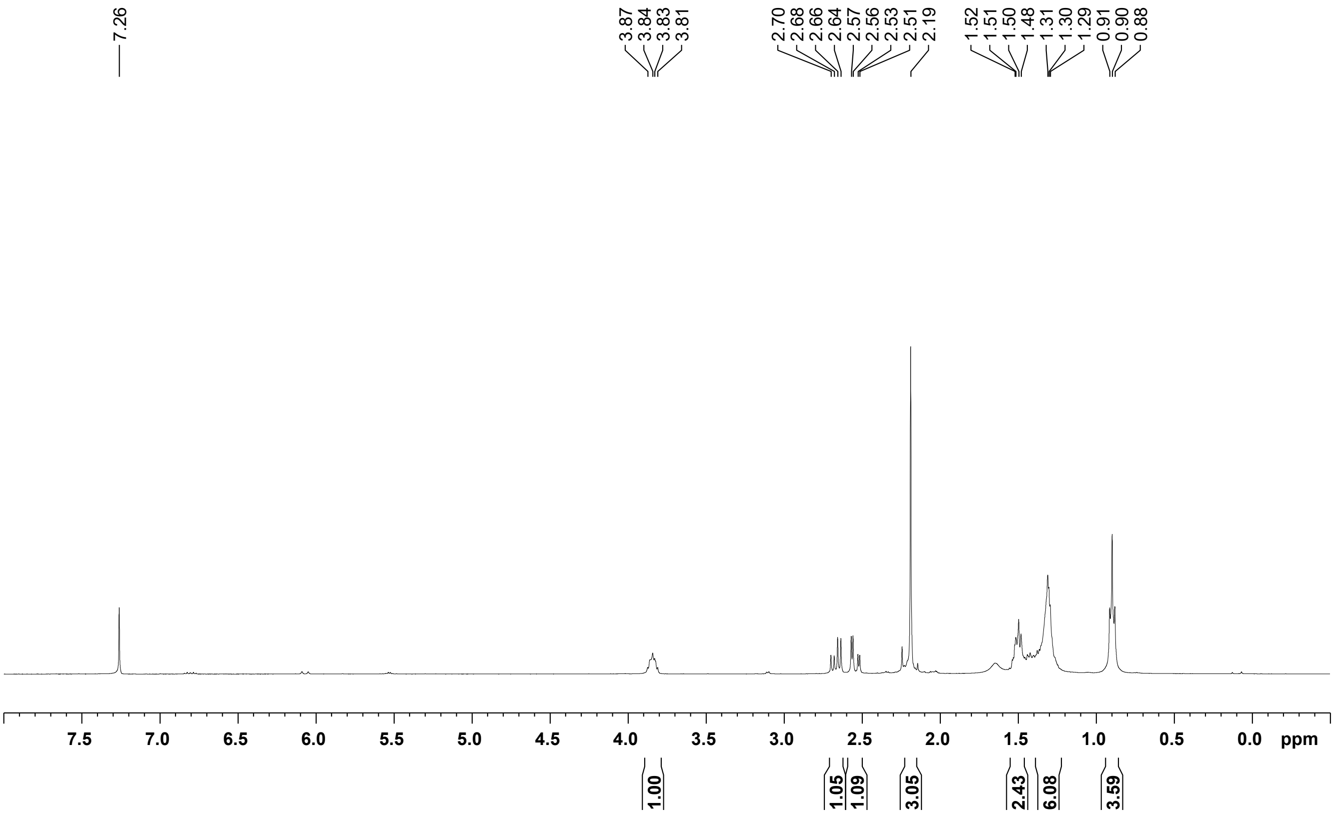


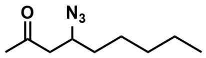


4-azidononan-2-one (**2b**)

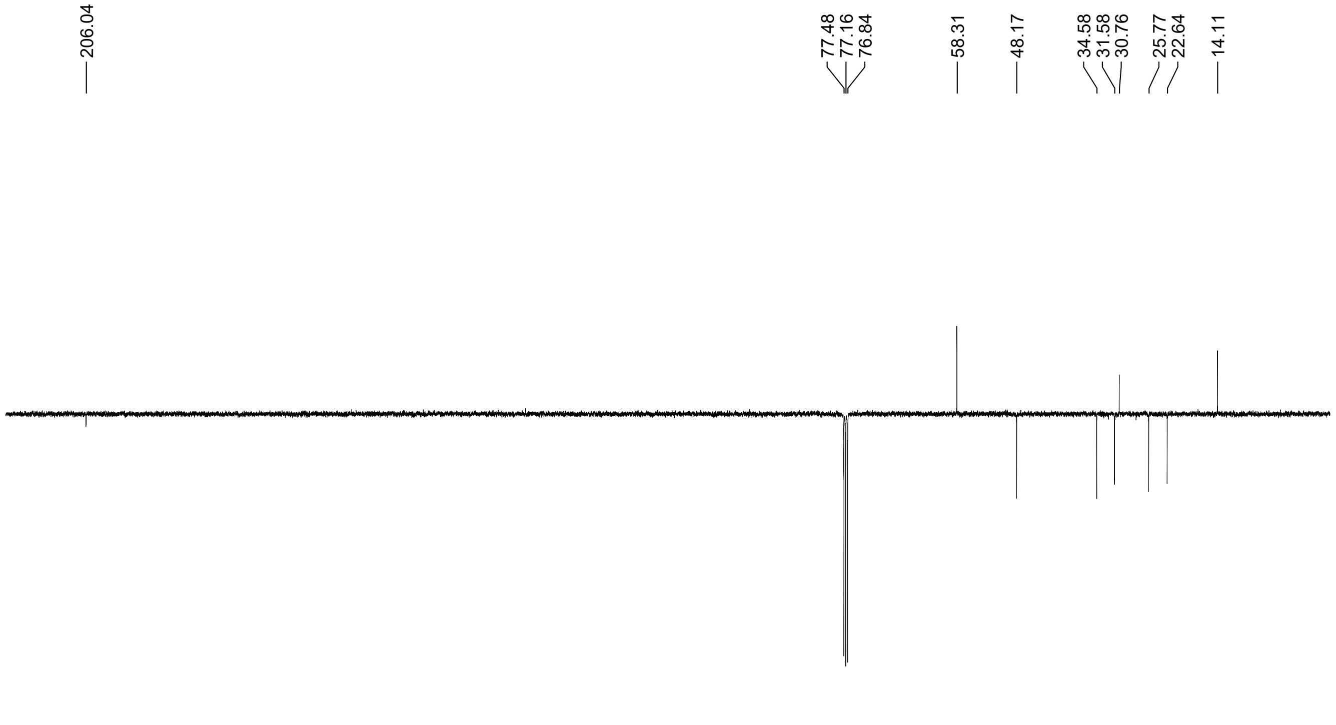

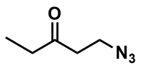


1-azidopentan-3-one (**2c**)

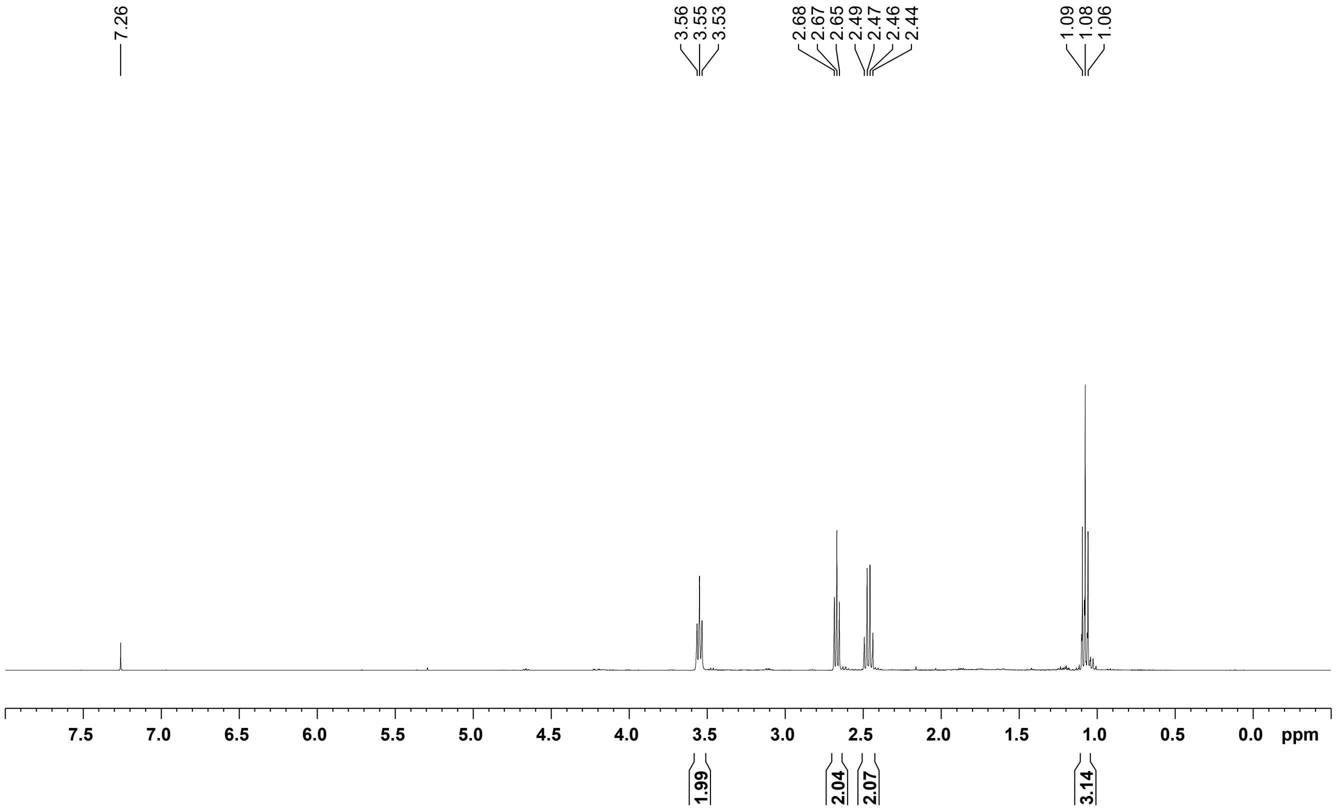


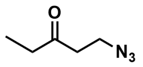


1-azidopentan-3-one (**2c**)

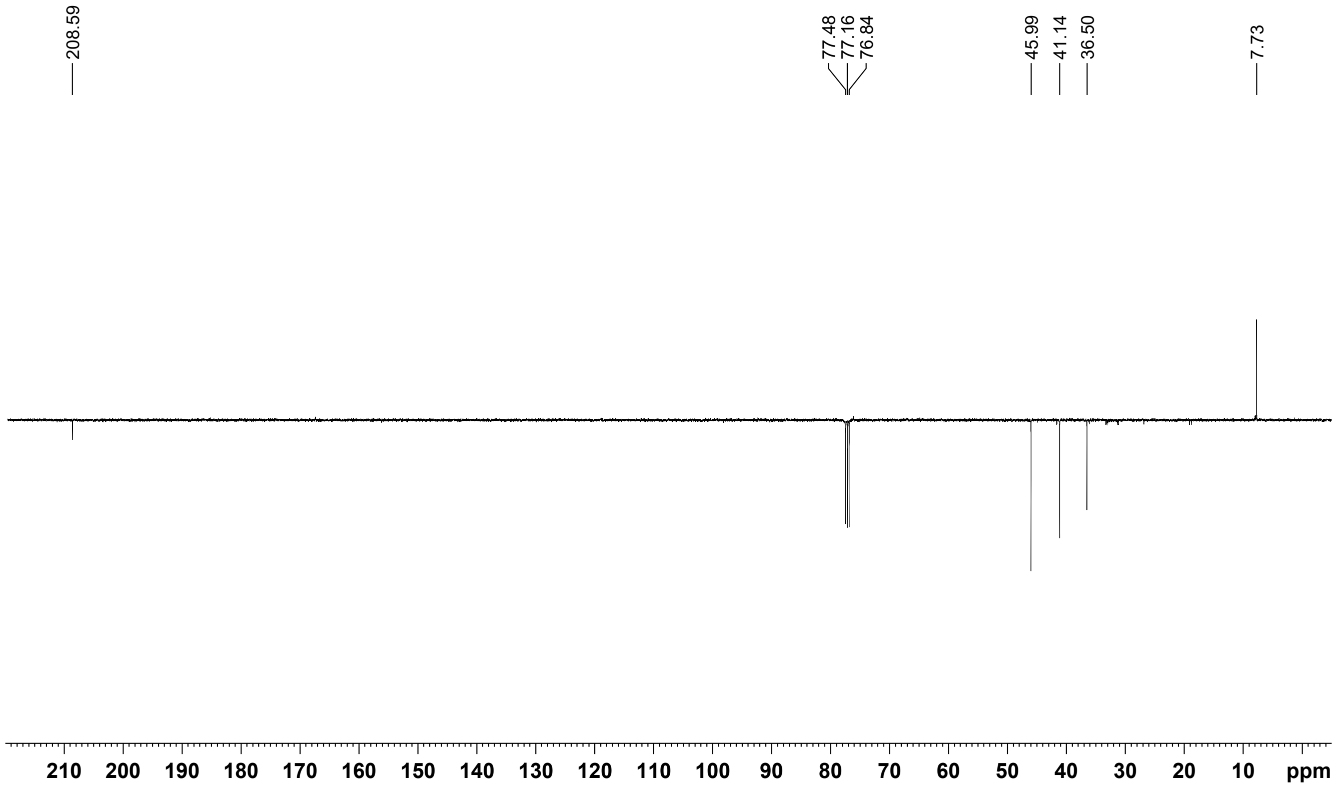

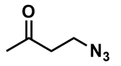


4-azidobutan-2-one (**2d**)

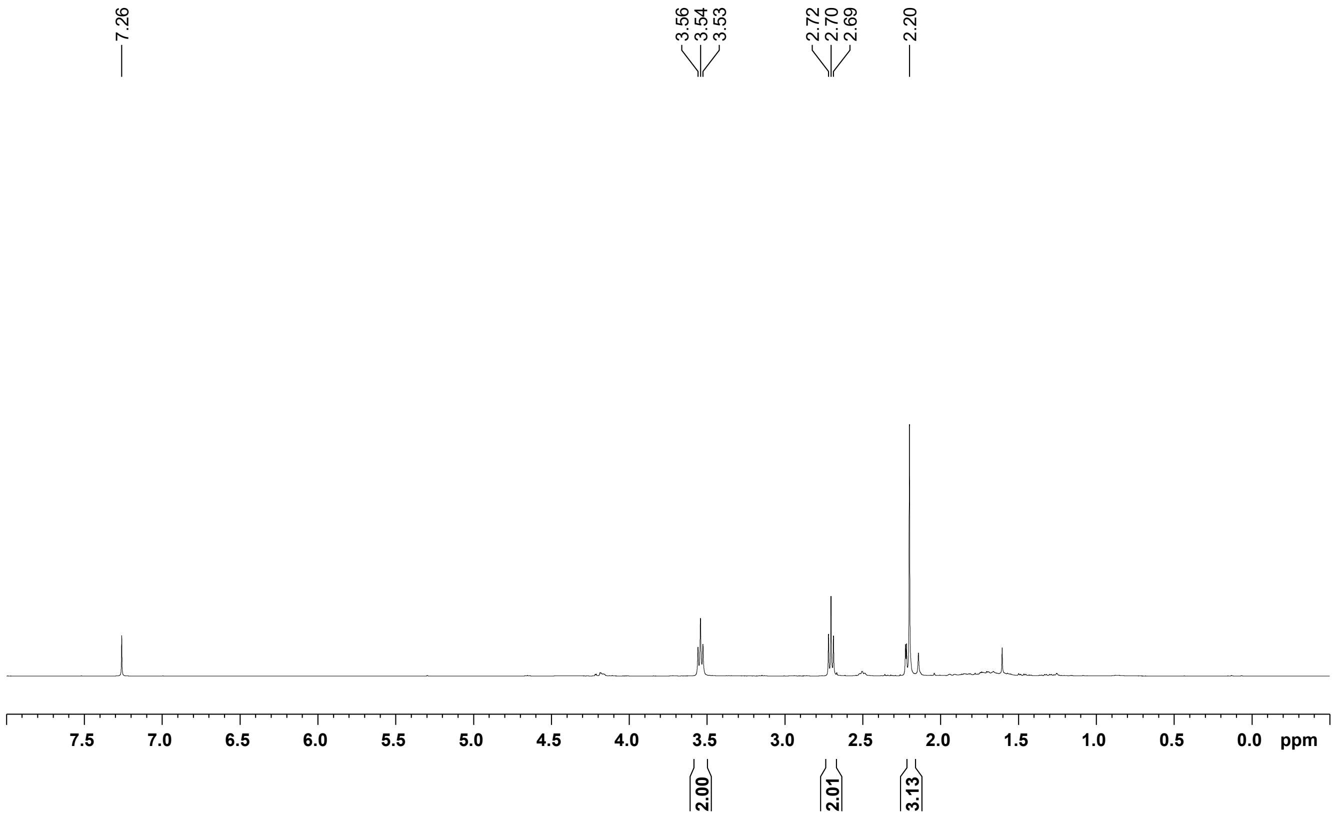


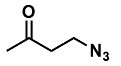


4-azidobutan-2-one (**2d**)

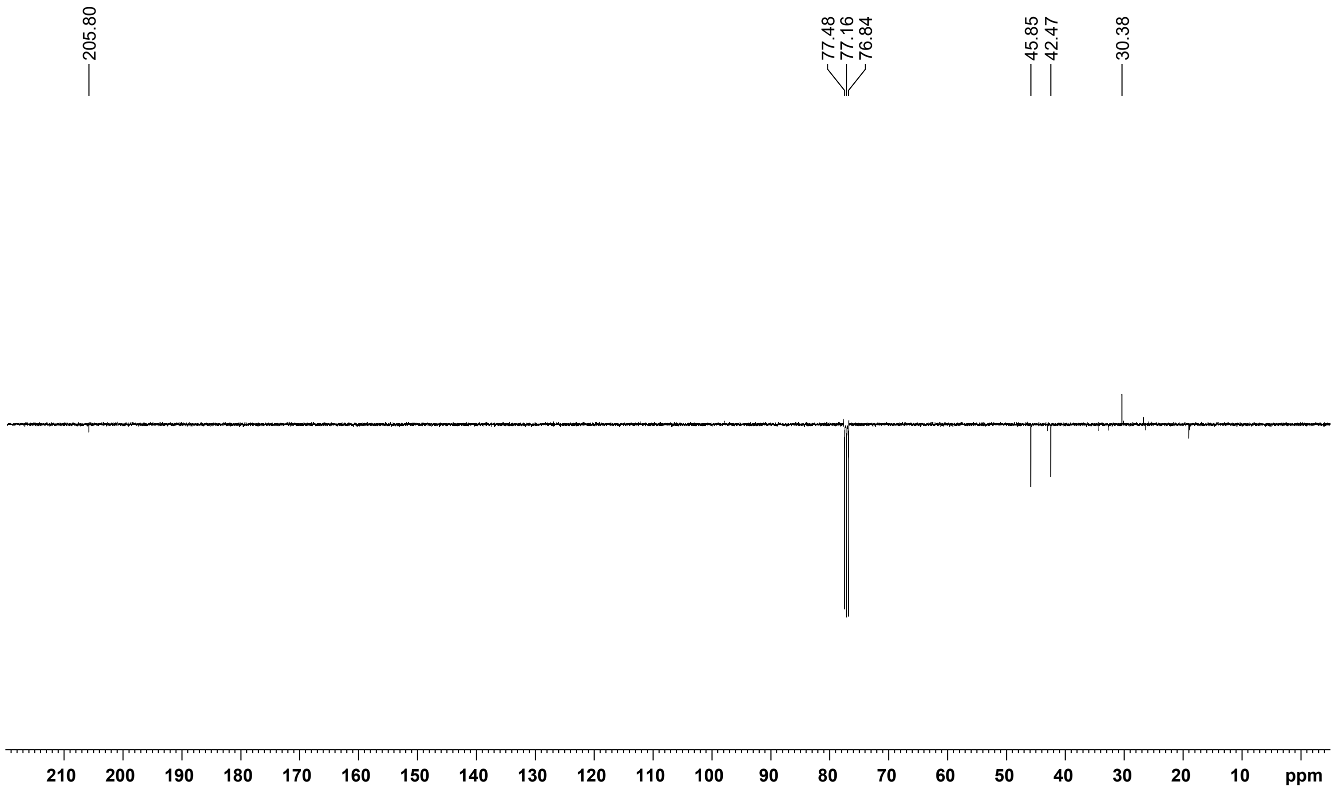

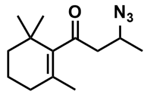


3-azido-1-(2,6,6-trimethylcyclohex-1-en-1-yl)butan-1-one (**2e**)

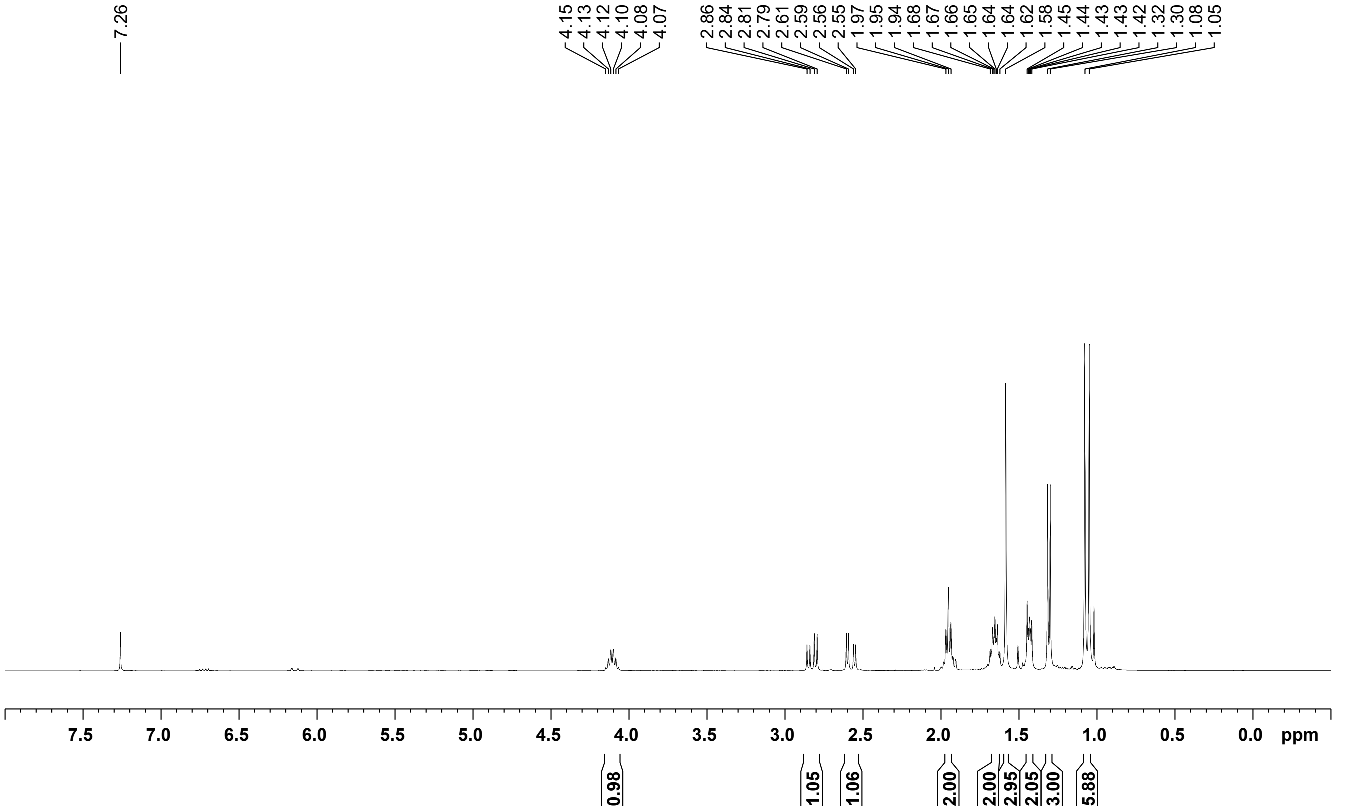


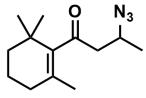


3-azido-1-(2,6,6-trimethylcyclohex-1-en-1-yl)butan-1-one (**2e**)

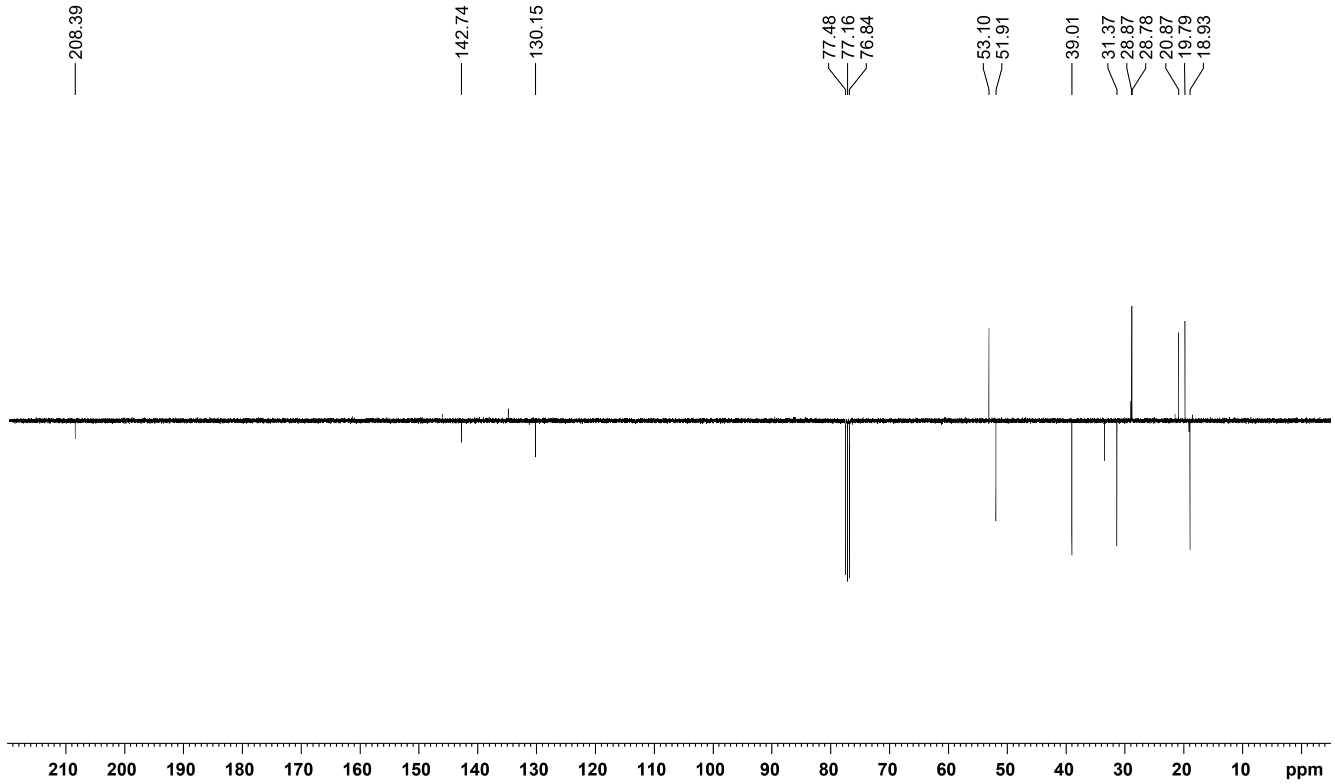

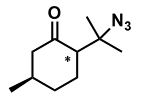

(5R)-2-(2-azidopropan-2-yl)-5-methylcyclohexanone (**2f**)

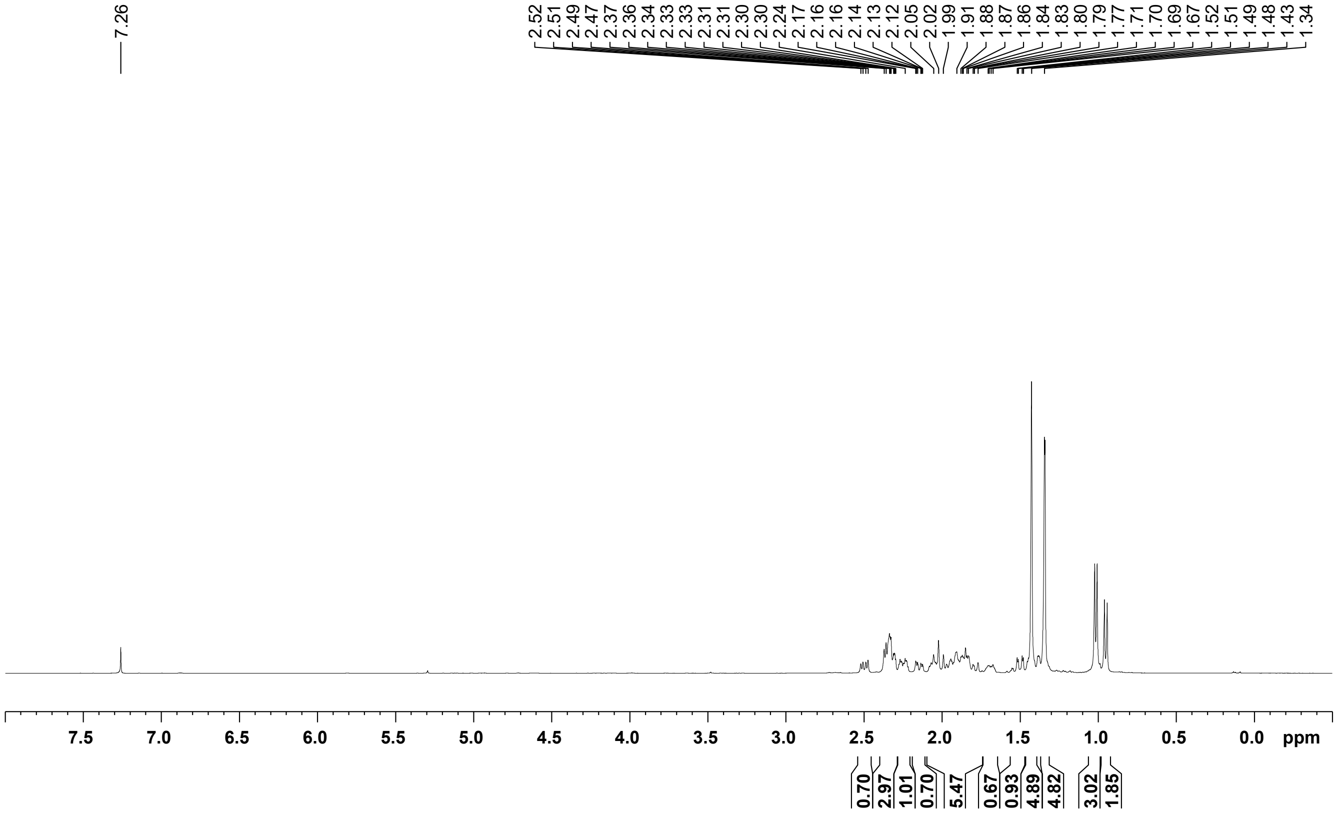


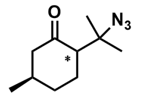

(5R)-2-(2-azidopropan-2-yl)-5-methylcyclohexanone (**2f**)

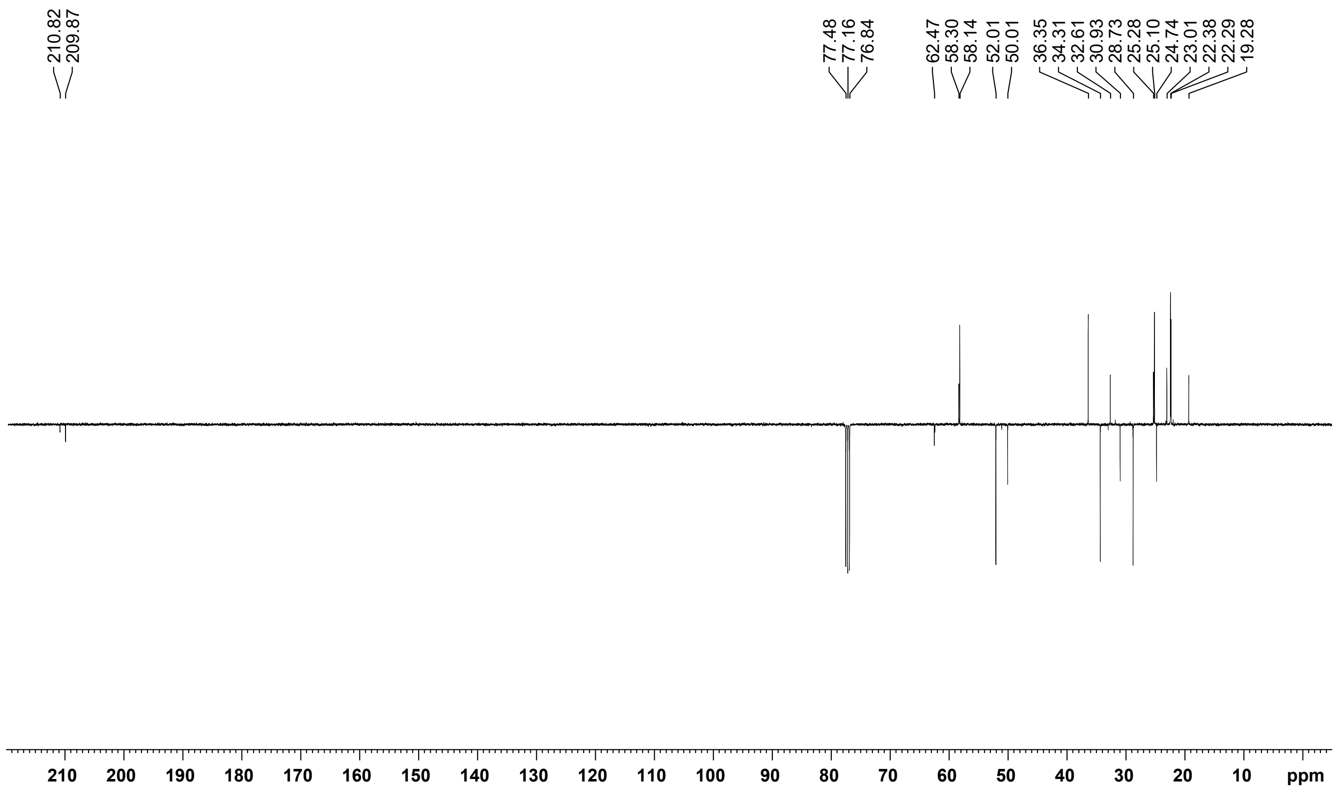

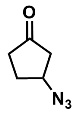


3-azidocyclopentanone (**2g**)

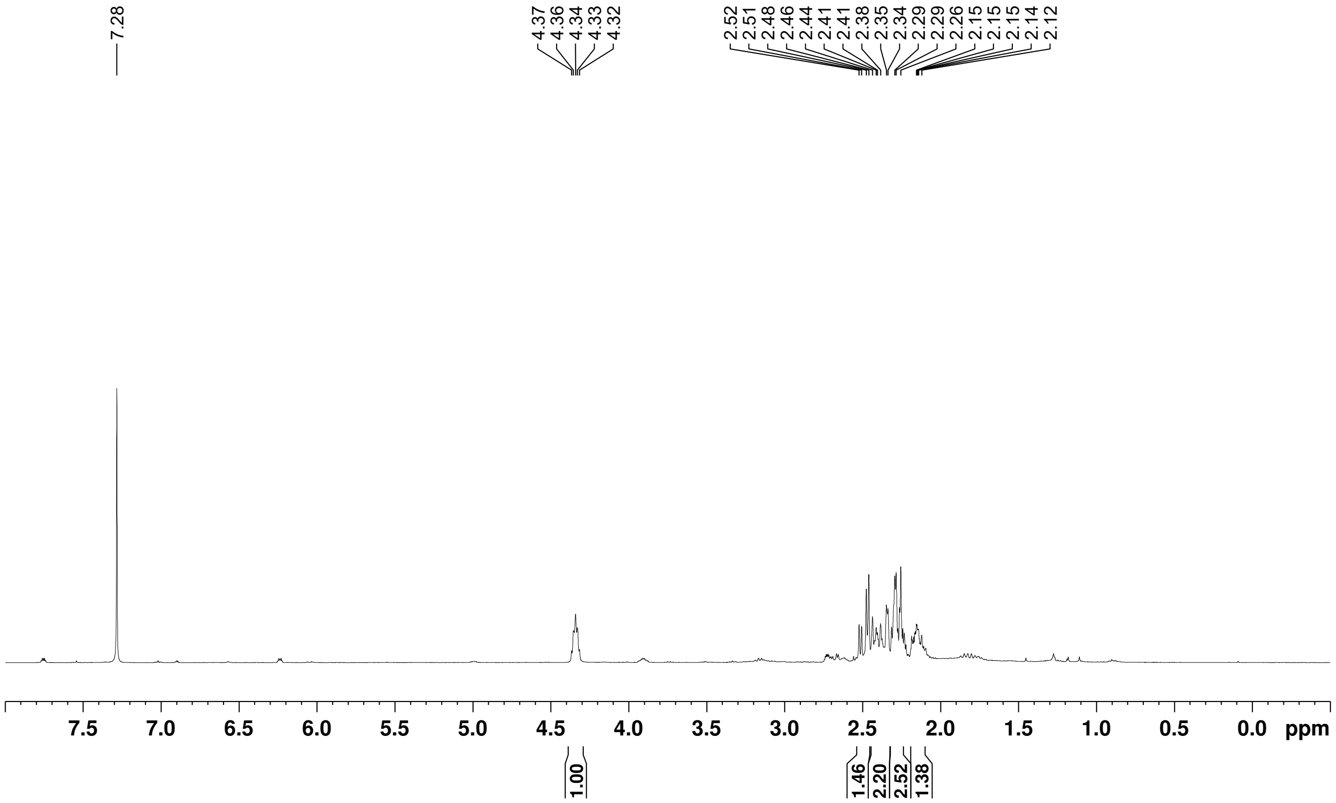


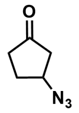


3-azidocyclopentanone (**2g**)

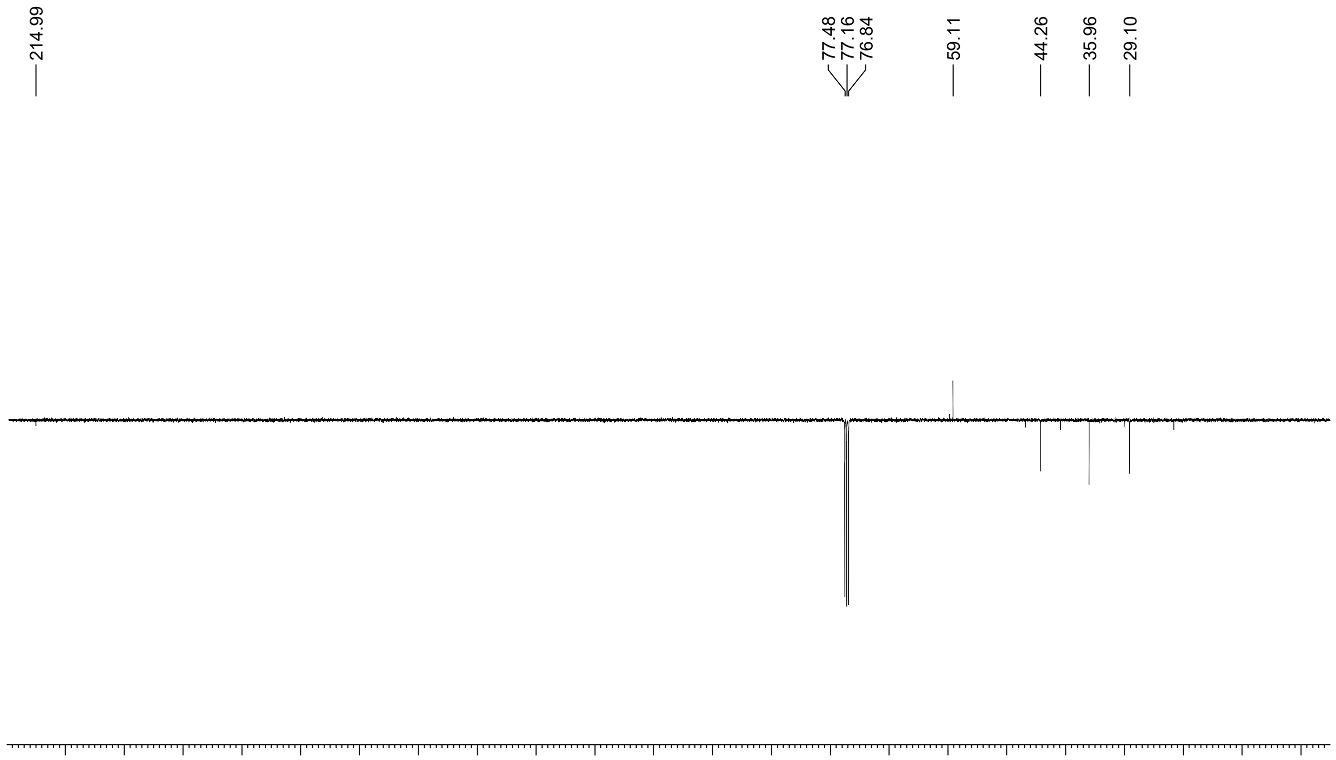

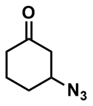

3-azidocyclohexanone (**2h**)

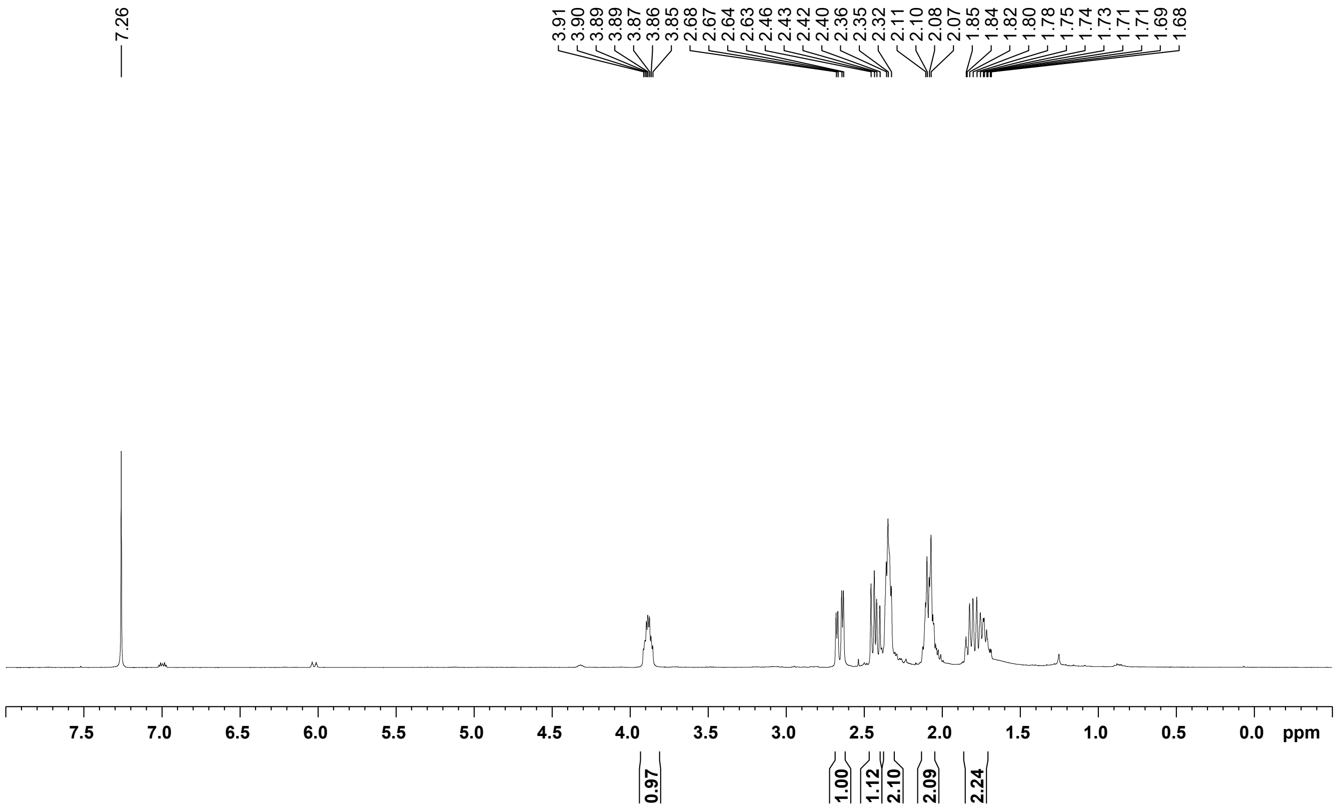


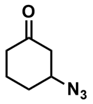

3-azidocyclohexanone (**2h**)

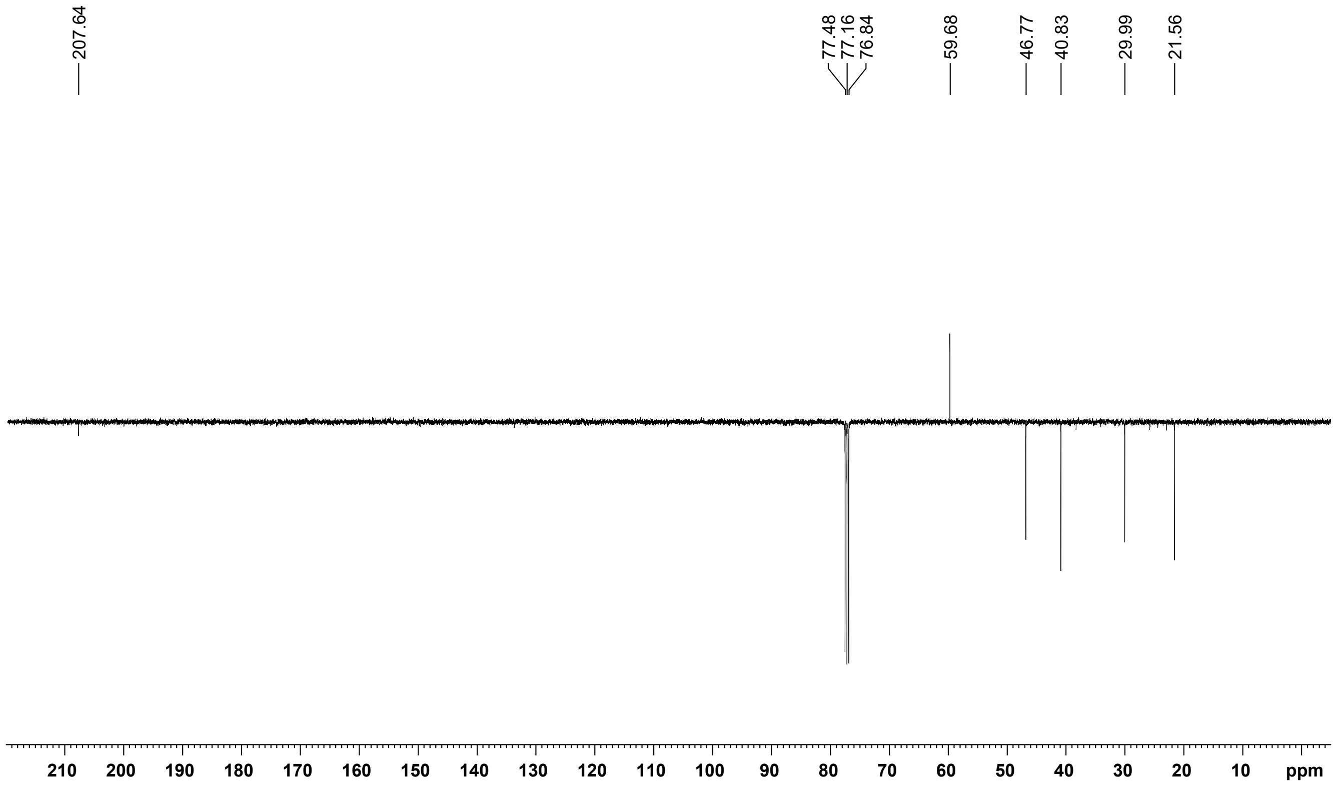

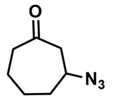


3-azidocycloheptanone (**2i**)

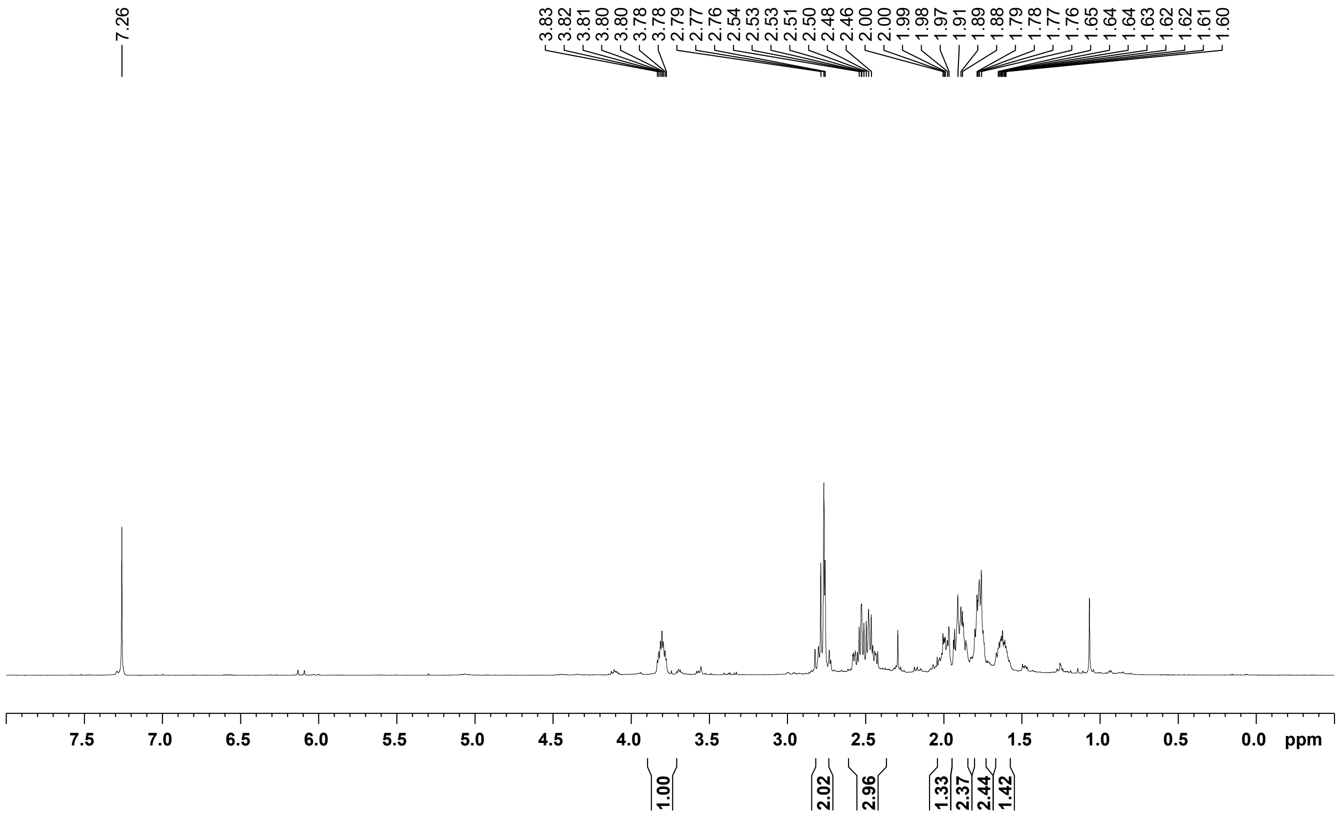


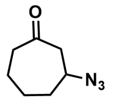


3-azidocycloheptanone (**2i**)

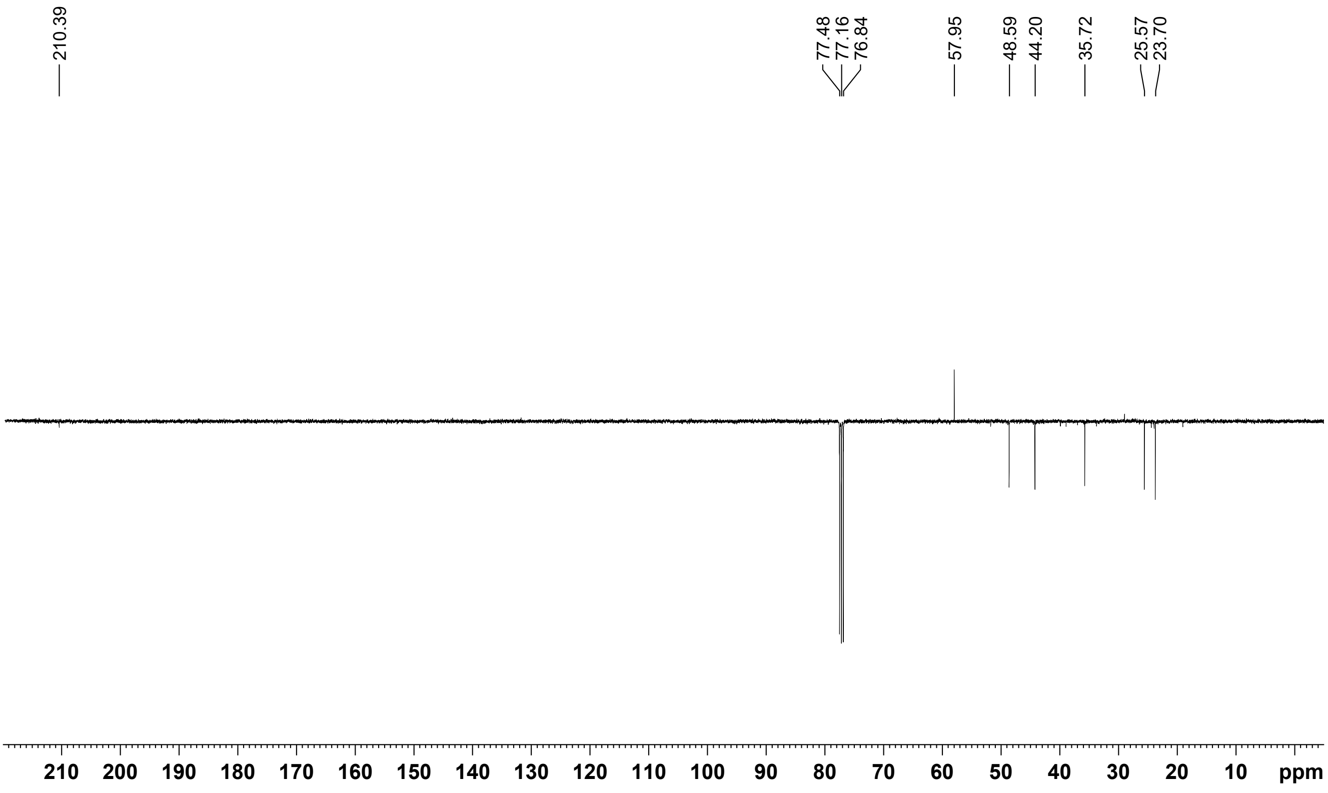

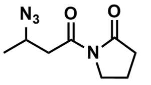


1-(3-azidobutanoyl)pyrrolidin-2-one (**2j**)

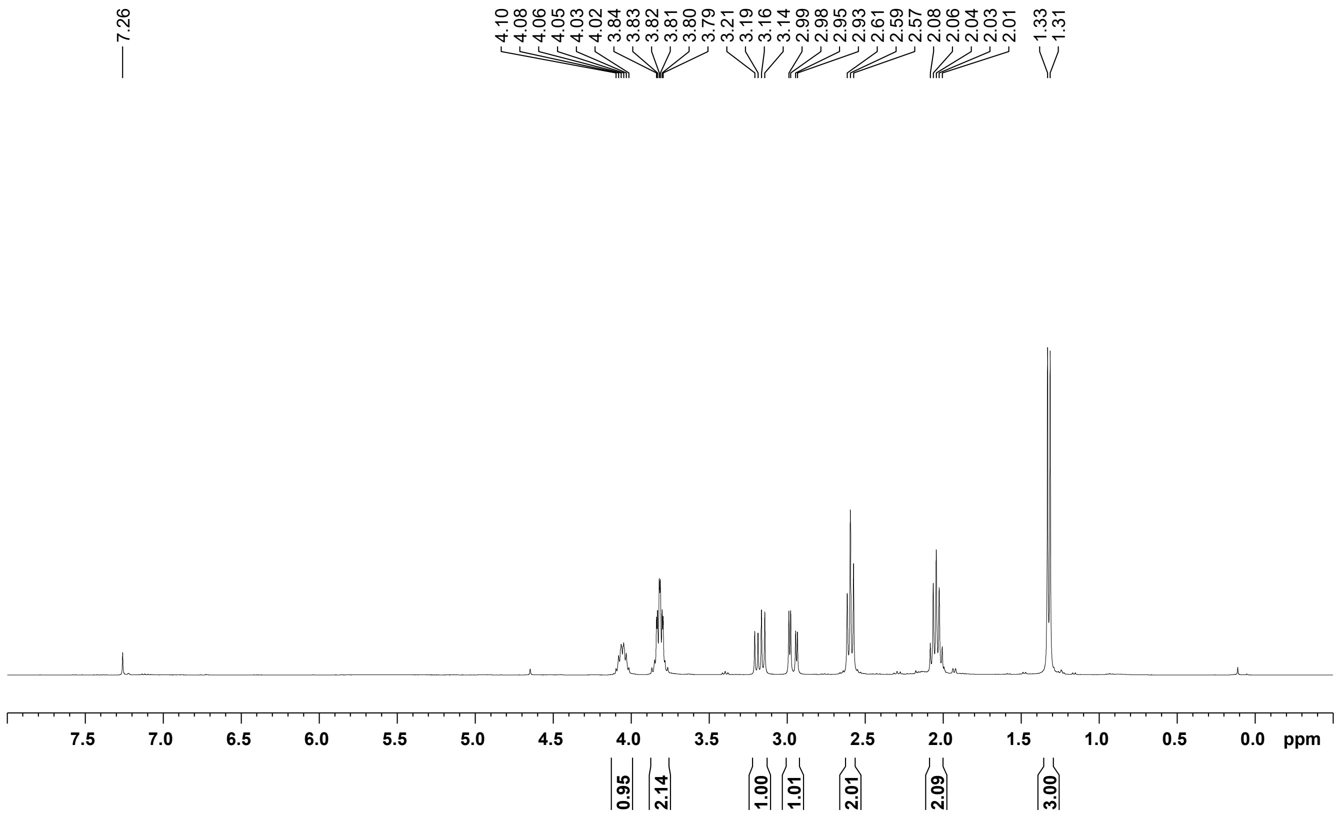


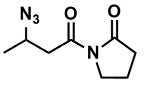


1-(3-azidobutanoyl)pyrrolidin-2-one (**2j**)

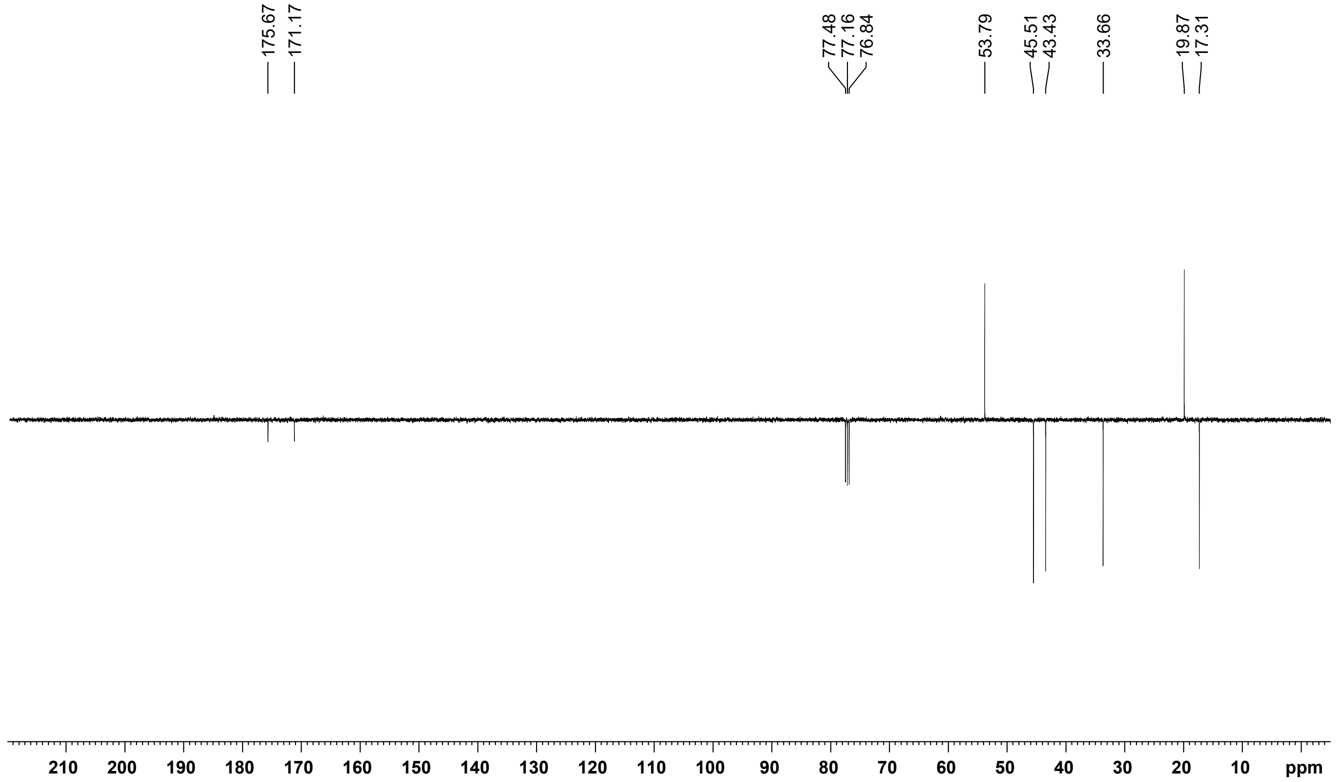

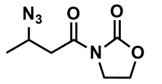


3-(3-azidobutanoyl)oxazolidin-2-one (**2k**)

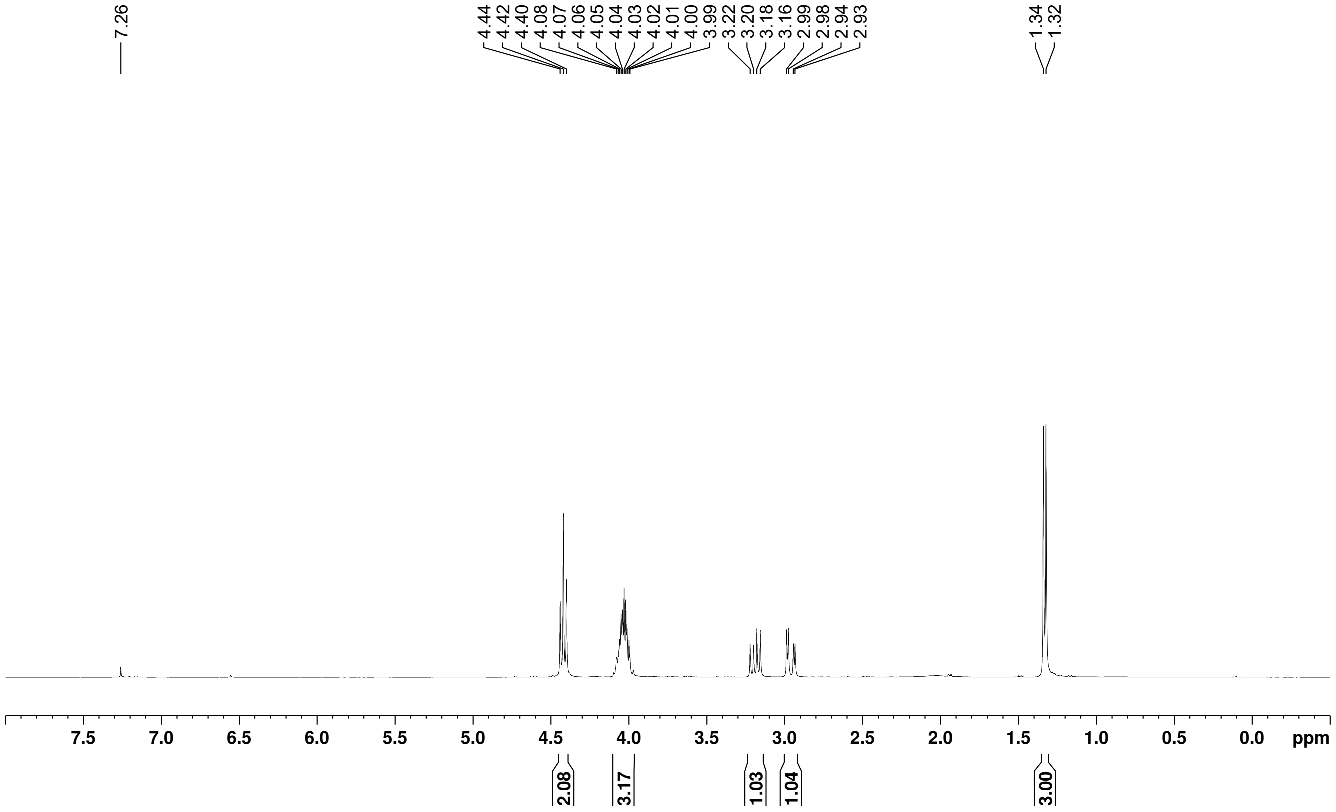


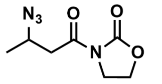


3-(3-azidobutanoyl)oxazolidin-2-one (**2k**)

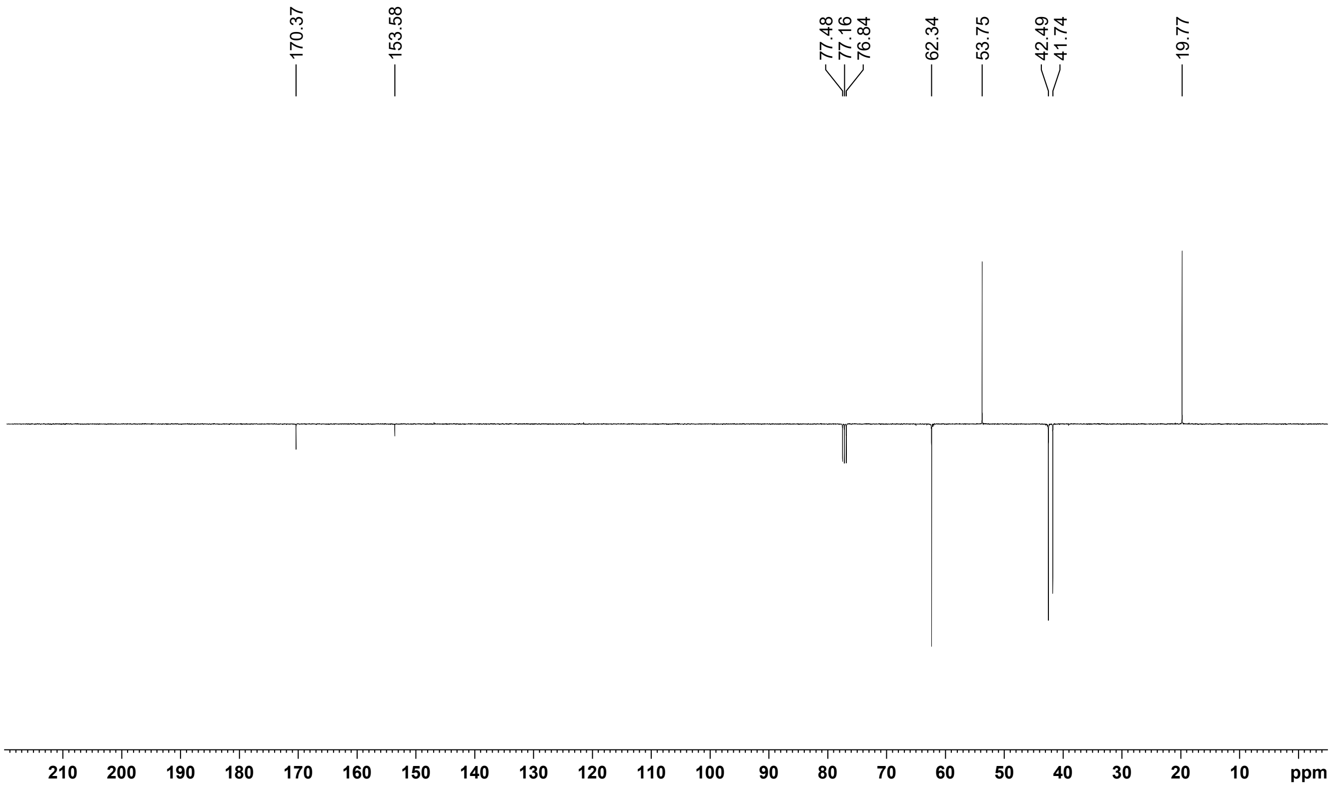

Supplement: Supplementary file 1 — sc2c07213_si_001.pdf [file sc2c07213_si_001.pdf]
